# Workplace Health and Wellbeing in Small and Medium Sized Enterprises (SMEs): A Mixed Methods Evaluation of Provision and Support Uptake

**DOI:** 10.3390/ijerph22010090

**Published:** 2025-01-10

**Authors:** Nigel Lloyd, Nigel Smeeton, Imogen Freethy, Julia Jones, Wendy Wills, Abi Dennington-Price, John Jackson, Katherine Brown

**Affiliations:** 1 School of Life and Medical Sciences, University of Hertfordshire, Hatfield AL10 9AB, UK; i.freethy@herts.ac.uk (I.F.); j.jackson21@herts.ac.uk (J.J.); k.brown25@herts.ac.uk (K.B.); 2 Centre for Research in Public Health and Community Care (CRIPACC), School of Health & Social Work, University of Hertfordshire, Hatfield AL10 9AB, UK; n.smeeton@herts.ac.uk (N.S.); j.jones26@herts.ac.uk (J.J.); w.j.wills@herts.ac.uk (W.W.)

**Keywords:** workplace wellbeing, SME, public health, small and medium sized enterprises, barriers and facilitators, health and wellbeing support, workplace health promotion, mixed method, health and wellbeing

## Abstract

Today’s challenging times highlight the need for workplaces to support employee wellbeing. Workplaces can offer a means to improve employee wellbeing and promote health initiatives. However, small and medium-sized enterprises (SMEs) are less likely than larger organizations to engage with workplace wellbeing initiatives or offer wellbeing provision. This study, conducted in an urban area in central England, explores SME engagement with local government workplace wellbeing provision, and barriers and facilitators to SME engagement, SME implementation of wellbeing provision, and employee uptake. A mixed-methods design was used. Quantitative data were collected via a survey of 103 SMEs and qualitative data from three focus groups with stakeholders involved in promoting SME engagement with wellbeing support (n = 9) and 16 in-depth interviews with SME representatives (n = 8) and employees (n = 8). Quantitative data were analyzed using the chi-squared, Fisher’s exact and Mann-Whitney U tests, and multivariable logistic regression. Qualitative data were analyzed using framework analysis. Findings highlighted several interrelated factors acting as barriers and facilitators to SME engagement with wellbeing initiatives, SME-provided wellbeing provision, and employee uptake. The study provides valuable insights for policymakers, public health teams, and SME leaders on improving provision of and engagement with wellbeing programs. Trust, awareness, knowledge, and communication are highlighted as important prerequisites of optimal provision and engagement.

## 1. Introduction

### 1.1. Background

#### 1.1.1. Health and Wellbeing and Work—A Growing Area of Interest

A significant proportion of working age adults’ (between 16 and 64 years old) waking hours are spent at work or on work-related tasks, with estimates varying from around 20 per cent to more than half [1,2]. Consequently, health and wellbeing in the workplace have been the focus of considerable research activity. The extent and nature of employee health and wellbeing, the factors influencing employee health, and the influence of wellbeing on productivity are among the common areas of interest. Recent shifts in the nature of working life and changing global patterns of health and wellbeing have contributed to heightened government, employer and academic interest in the topic of workplace wellbeing [3,4,5,6].

Non-communicable diseases (NCDs) now account for more than 70 per cent of deaths globally, with cardiovascular disease (CVD) the leading global cause of death, and cancers, chronic respiratory diseases and diabetes the other main contributors to NCD mortality [7]. The increasing prevalence and earlier onset of such diseases, means they are increasingly affecting people of working age [8]. Promoting workplace wellbeing is important as it influences both non-work lives and productivity [3,9]. Workplaces also provide a platform for disseminating public health and health improvement messages and interventions [10].

In addition to the changing global health context, disparities in health equity [11,12], increasing economic costs related to ill-health [13] and a growing appreciation of the multifaceted and holistic nature of health and wellbeing [14,15], have helped to generate interest in this area. The COVID-19 pandemic has also spurred interest. Changing modes of working in some societies (e.g., the increase in home and teleworking) have created fresh challenges for worker wellbeing and necessitated new approaches to addressing these.

#### 1.1.2. Workplace Health and Wellbeing—The United Kingdom Context

In the United Kingdom (UK), employee health and wellbeing have been a focus for policy intervention as data suggest UK full-time workers spend amongst the highest number of hours at work of any European country [16]. Long working hours can result in individuals’ work and personal lives becoming heavily intertwined, and research suggests that the combination of work and everyday stressors can result in detrimental emotional and physical outcomes [17]. Indeed, in current times in the UK, supporting the wellbeing of employees is of particular importance. UK workers face various challenges, including the ongoing UK cost of living crisis fueled by high cost inflation, which research suggests, negatively impacts mental health [18], low levels of pay growth since the 2008 recession [19], and a growth in insecure work [20].

UK Labour Force Survey data indicate that UK sickness absence rates—the percentage of working hours lost because of sickness or injury—are increasing [21]. In 2022, the sickness absence rate was 2.6%, an increase from pre-pandemic levels of 1.9% in 2017 and 2.0% in 2018, and the highest level since 2004. An estimated 185.6 million working hours were lost to sickness or injury in the UK in 2022, a record high equating to more than five days lost per worker. All age groups experienced increases in their sickness absence rate in 2022 and women, older workers, and those with long-term health conditions, had the highest rates [21]. The economic cost of workplace injury and illness in 2022/2023 was estimated at £21.6 billion [22]. UK data suggest that mental wellbeing may be a particular issue for UK workers, with an estimated 50% of days lost to work-related ill-health resulting from stress, depression or anxiety [23].

The UK has also experienced a post-COVID increase in working-age people who are ‘economically inactive’, that is, out of work and not actively seeking employment. At the beginning of 2024, this number reached its highest level since 2012, with the key driver identified as long-term sickness, the incidence of which had been rising since late 2019 [24]. Tackling the high levels of sickness absence and economic inactivity, and supporting employees to stay well while in work, was an identified policy priority for the new UK Government in July 2024 [25]. Workplace wellbeing interventions that improve employees’ health and wellbeing are thus, a priority.

#### 1.1.3. Addressing Workplace Health and Wellbeing

Workplace health and wellbeing interventions may take several different forms. These include support for addressing work-related health problems, such as mental health disorders, back pain, and musculoskeletal disorders [26], as well as support that reflects a holistic view such as that reflected in the UK Chartered Institute for Personnel Development (CIPD) model of workplace health and wellbeing which includes reference to values and principles, collective and social relationships, and personal growth [27].

Various studies have identified the wellbeing benefits and return on investment that can result from health and wellbeing initiatives [28,29,30,31]. However, organizations differ in their approaches to workplace wellbeing and the extent of provision for their employees. In England, local authorities have a responsibility for providing public health services to local populations, and this often includes provision for local organizations to help support the health and wellbeing of their employees.

#### 1.1.4. Workplace Health and Wellbeing in SMEs

Small and medium sized enterprises (SMEs) are often a focus for local authority support, as smaller organizations are less likely to offer occupational health support than larger ones, or to engage with government workplace wellbeing support programmes [32]. SMEs are organizations with fewer than 250 employees. Various definitions of SMEs exist but for this study, we followed the approach of the United Kingdom Government’s annual longitudinal small business survey [33] and defined an SME solely in terms of its number of employees (0–249) [34]. SMEs account for the majority of businesses worldwide [35] and for 99.9% of the 5.6 million private sector businesses in the UK and employ approximately 16.3 million people, 61% of the total private sector employed population [36] and approximately 50% of the total UK employed population. Of significance, previous research suggests that those working within SMEs can be at greater risk of poor wellbeing at work than those in larger organizations [37,38,39]. A range of organizational factors may account for this, including high workloads, the need for SME employees to take on multiple work roles, and work/life imbalances [40]. In addition, encouraging SMEs to actively support the health and wellbeing of their staff can be particularly challenging since they may have limited time, financial resources, human capital, organizational commitment, or know-how to facilitate such support [41,42,43,44]. The large proportion of workers employed by SMEs, the specific wellbeing challenges faced by these organizations, and the current challenging business environment, mean that a focus on workplace health and wellbeing in SMEs is especially significant and timely.

### 1.2. The Current Study

This research was designed to evaluate the use of workplace health and wellbeing support by SMEs across an English local authority area and provide recommendations about how support might be optimized. The local authority encompasses an ethnically diverse urban area in central England, with a legacy of traditional manufacturing industry. The area has significant levels of deprivation, higher unemployment rates than the national average, and a high proportion of the population at risk of a range of preventable health conditions [45,46].

The local authority provided a workplace health program (WHP) for SMEs that was free at the point of use (also known as a WHISPA [46]). The WHP, which had been operational for approximately seven years at the time of our research, provided a suite of services which SMEs could access depending on their requirements. It was delivered by an independent not-for-profit organization and aimed at SMEs with between 10 and 249 employees. It included:

- Delivery of National Health Service (NHS) health checks [47] and health assessments for SME staff;
- Advice and support for SMEs in assessing staff wellbeing needs;
- Support to gain regional workplace wellbeing accreditation;
- Provision of health and wellbeing workshops for staff;
- A 12-week behaviour change support programme for individual staff members;
- Support for SMEs to develop workplace wellbeing polices.

This study aimed to explore the extent and nature of SME engagement with the WHP, and barriers and facilitators to that engagement. We also sought to explore barriers and facilitators to implementation of workplace wellbeing provision for SME employees, and to employee uptake. The objectives of the study were to explore:

1. Barriers and facilitators to SME engagement with the WHP;
2. SMEs’ motivations for (a) accessing the WHP; (b) provision of workplace health and wellbeing support to their employees;
3. The factors influencing SMEs’ provision of workplace health and wellbeing support for their employees;
4. Barriers and facilitators to SME employees’ engagement with employer-provided workplace health and wellbeing provision.

To our knowledge, this is the first mixed method study to simultaneously explore SME engagement with external support, SME wellbeing provision for employees, and employee uptake of provision. Findings will have relevance to those aiming to enhance future SME workplace health and wellbeing provision.

## 2. Materials and Methods

### 2.1. Design

This study employed mixed methods design, incorporating analysis of primary quantitative and qualitative data. Quantitative data were collected via online or telephone survey. Qualitative methods were focus groups and semi-structured in-depth interviews. The mixed-method approach enabled us to address our research objectives/questions more comprehensively than using either approach alone. It also allowed for triangulation of findings [48] and complementarity in the analysis process (using results from one method to enhance, elaborate or clarify findings from the other) [49]. Our qualitative analysis is underpinned by a broad constructivist/interpretivist orientation [50], acknowledging the role of individuals’ experiences and interpretations in the framing of their subjective, constructed realities.

Approaches to integrating qualitative and quantitative research procedures and data can be implemented at ‘design’, methods’, and ‘interpretation and reporting’ stages of research [51]. For this study, qualitative and quantitative data have been integrated at the ‘interpretation and reporting’ level, with each type of data separately analyzed and then synthesized [52]. As this was an exploratory study, conducted on behalf of the local authority, no a priori theoretical framework was used in the coding and analysis of data. Consolidated Criteria for Reporting Qualitative Research (COREQ) reporting guidelines [53] were applied in reporting the qualitative study components.

### 2.2. Ethical Considerations

Ethical approval for the study was granted by the University of Hertfordshire Health, Science, Engineering, & Technology Ethics Committee with Delegated Authority (ECDA): HSK/SF/UH/04929. Participants were provided with full study details prior to consenting and were assured of their anonymity and compliance with General Data Protection Regulation (GDPR).

### 2.3. Patient and Public Involvement and Engagement (PPIE)

PPIE was an integral part of our work. Despite challenges, we successfully recruited one local resident by working with a local charity. The local contributor was involved in reviewing the survey questionnaire, developing ideas for recruiting local SME employees and commenting on evaluation findings. Wider lay/public involvement and scrutiny was achieved throughout the project via our team’s Public Involvement in Research group (PIRg). They are a diverse group of ten individuals, located across the UK, which meets monthly, and is chaired by our team’s public co-investigator. Two members of the PIRg were embedded in the research team and supported development of the project from inception to completion. Their work included: refining the research questions and scope; commenting on the protocol; attending regular project team and advisory group meetings; co-developing the evaluation approach; co-designing the survey questionnaire; supporting data analysis (including qualitative framework analysis and quantitative analysis); co-designing project outputs; and co-authorship of this manuscript.

### 2.4. Participants

Three participant groups took part: stakeholders involved in encouraging SME engagement with workplace health and wellbeing support, SME employers, and employees of SMEs. Since the WHP support for SMEs was aimed primarily at organizations sized 10–249, micro-organizations (those with 0–9 employees), were excluded.

#### 2.4.1. Participant Group 1: Stakeholders Involved in Encouraging SME Engagement with Workplace Health and Wellbeing Support

##### Participant Group 1: Recruitment

The research team worked with council partners to identify potential participants working within the council and partner organizations, who had been involved in encouraging SME engagement with workplace wellbeing support during the previous three years. Potential participants were contacted via email and invited to take part in an online focus group discussion. A secure online Research Electronic Data Capture (REDCap) site [54] was used to present participant information and gain e-consent.

##### Participant Group 1: Data Collection—Focus Groups

Three focus groups were conducted with a total of nine stakeholders. Participants were: staff delivering the WHP (n = 2); local authority staff delivering workplace health and wellbeing support (n = 5); and representatives from partner organizations supporting SMEs with workplace wellbeing (n = 2). Focus groups took place between April and May 2022 and lasted between 61 and 75 min.

#### 2.4.2. Participant Group 2: SME Employers

##### Participant Group 2: Recruitment

Representatives of SMEs included business owners, chief executive officers (CEOs), managing directors, and human resource managers and were involved in two data collection methods: a survey (telephone or online) and semi-structured in-depth interviews. At the time of recruitment, local business intelligence data suggested there were approximately 900 SMEs with 10–249 employees in the local area. Discussions with local partners indicated that sample sizes gained via surveys of local businesses were typically small. We therefore aimed for a total survey sample size of 75. On this basis, only a descriptive analysis was planned, and an a priori power analysis was not performed. However, the precision of estimates was considered; a sample of size 75 allows an estimate of a proportion to be correct to ±12%.

An independent research organization was commissioned to administer a telephone survey of SMEs, using a commercially available list of local SMEs sized 10–249 employees as the sampling frame. The team delivering the WHP and other local partners also assisted SME recruitment by sharing evaluation information and a link to an online version of the questionnaire during their routine communications with SMEs. Using the link, participants were able to read the participant information, provide e-consent, and complete the online survey (survey questionnaire provided in Appendix A). To encourage participation, SMEs were informed that for every questionnaire completed, a donation would be made to a local charity. In total, 103 survey questionnaires were completed by SMEs (100 telephones and three online). A range of SMEs were represented. Table 1 provides details of the size and industrial sector of SMEs in our sample and the locality (Table 1).

Recruitment of SMEs for in-depth interviews was conducted using both purposeful and snowball sampling. All survey respondents were invited to register to participate in an interview. SMEs were also recruited through contact lists held by the local authority and partner organizations, and via research team attendance at local business events. Interview participants also identified other potential participants. The research team used a commercially available list of local businesses to identify and recruit local SMEs. Eight interviews were conducted with representatives from SMEs. Table 2 details the number recruited via each method.

##### Participant Group 2: Data Collection—Survey

Questionnaire development

The research team developed the survey questionnaire in collaboration with local authority partners, staff delivering the WHP, and public contributors, all of whom provided feedback and comments over a three-month period.

We aimed to develop a concise questionnaire to maximize the likelihood of completion [55], and to collect information that was both of value for local services, and that would inform SME workplace health and wellbeing practice more widely. A draft version of the survey was piloted with a SME employer and a local organization supporting SMEs with workplace health and wellbeing, and revisions were made. Table 3 provides details of the topics covered.

Survey completion

The telephone survey took place from June to July 2022, with the online survey simultaneously available. The commissioned research organization conducted the telephone survey using versions of the study materials adapted for telephone administration. A total of 1167 SMEs were contacted by telephone, with a maximum of five attempts made to contact an organization. A total of 100 telephone interviews were conducted, each taking approximately 15 min. Three participants completed the survey online (of approximately 30 SMEs invited to participate this way). Table 4 details the roles of those who participated on behalf of their SME.

##### Participant Group 2: Data Collection—Semi-Structured Interviews

Eight individual, semi-structured interviews were conducted with representatives from SMEs. Interviews took place remotely (via videoconferencing or telephone) between July 2022 and January 2023, and lasted between 15 and 44 min. Topics explored included SMEs’ experiences of accessing workplace health and wellbeing support; gaps in available support; barriers to engagement with support and how these could be overcome; SMEs’ provision of wellbeing services; and perceptions of employee health and wellbeing needs. Table 5 presents the characteristics of SMEs represented in interviews.

#### 2.4.3. Participant Group 3: SME Employees

##### Participant Group 3: Recruitment

To avoid gatekeeping sampling biases that can occur when recruiting individuals from within organizations [56], we sought primarily to recruit employee participants through a range of routes that did not include direct recruitment through employing organizations. These routes were:

- WHP team members registering employees’ interest in participation during health check visits to SMEs (employees were invited to provide their contact details via a REDCap site and were later contacted by the research team);
- recruitment via local community and not-for-profit organizations’ contacts;
- publicity in local venues, such as libraries and community centres;
- awareness-raising by council teams and community partners through their routine engagement with community members;
- outreach at local sporting events;
- postings on social media sites;
- snowball sampling.

Participants either read participant information and provided e-consent via an online REDCap form or a member of the research team read a shortened version to them, and they provided verbal informed consent. A ‘thank you’ shopping voucher was offered to all participants.

A total of eight employees participated in an in-depth interview. Table 6 provides further details.

##### Participant Group 3: Data Collection—Semi-Structured Interviews

Interviews took place remotely via videoconferencing software (one interview) or telephone (seven interviews) and lasted between 18 and 47 min. Interviews were conducted between September 2022 and January 2023. They explored participants’ views and experiences of the workplace health and wellbeing provision available via their employers, and perceptions of any current gaps.

### 2.5. Quantitative Analysis

SME engagement with workplace wellbeing support was examined in terms of each of the four wellbeing support services available locally. The outcomes of interest were whether use was made of the resource by the SME during the previous 12 months and if not, the reasons for not using the resource during that period (options were: did use/did not need/not aware/not sure what support would involve/not enough time/other reason/don’t know or not sure).

Workplace wellbeing practice and support offered were analyzed based on the existence of an agreed workplace health and wellbeing strategy or plan, whether employees were consulted about their health and wellbeing needs, and the support or training made available to employees in the previous 12 months. Types of support analyzed were health and safety, stress management/reduction, mental health, injury prevention, stopping smoking, drugs/alcohol harm awareness, healthy lifestyles, ageing well, an employee assistance programme, financial management/health, caring responsibilities, and line management.

SME needs regarding workplace health and wellbeing support were assessed based on participant-reported interest in receiving various types of support for its workforce (identifying health/wellbeing needs of staff; NHS health checks/assessments; wellbeing workshops; support with developing a staff wellbeing survey; smoking cessation; support around drink or drugs; mental health awareness; support with healthy ‘behaviour change’ for staff; improving workplace health and safety; and developing workplace wellbeing policies).

SME views and attitudes to workplace health and wellbeing were analyzed using responses (on a 5-point Likert scale ordered from “strongly agree” to “strongly disagree”) to the first item of the 10-item scale: “Employers have a responsibility to support the health and wellbeing of employees”.

Explanatory variables (Table 7) were drawn from the background information section of the questionnaire. For time trading and category of enterprise the original options were merged due to small frequencies.

As more than 100 participants were recruited, the use of multivariable methods was considered appropriate [57]. Outcome variables with two categories were analyzed by multivariable logistic regression [58]. Outcome variables with three or more categories were modelled by merging categories to create a binary variable and applying multivariable logistic regression. Responses to the selected questionnaire item were analyzed using ordinal logistic regression. Findings were recorded as odds ratios, with associated 95% confidence intervals and *p*-values.

The modelling assumptions for logistic regression were examined. To determine whether this technique was appropriate, the linearity of the logit link was tested by the method described in Pregibon (1980) using the linktest command in Stata [59]. Multicollinearity was addressed by examining the pairwise correlations between the explanatory variables for large positive or negative values. Observations from SMEs were assumed to be mutually independent as only one individual was recruited from each participating enterprise. Outliers were identified from the residuals of the observations following model fitting. The degree of influence exercised by any outliers was assessed by reanalyzing the data following their removal.

For modest sample sizes, as in this study, modelling using all explanatory variables can lead to confidence intervals that are too wide to be meaningful [58]. To avoid this, explanatory variables first underwent a univariate selection procedure. For each categorical outcome variable, explanatory variables were taken in turn and either a chi-squared test or Fisher’s exact test applied. Responses to the questionnaire item were analyzed by applying the Mann-Whitney U test to each explanatory variable in turn.

Explanatory variables having *p* < 0.1 at the first stage were selected for entry into a logistic regression model. Choice of *p*-value in this process is discretionary [58], and 0.1 was chosen to keep the number of explanatory variables manageable given the sample size.

Not all participants gave a definitive response to each question, e.g., for certain questions some indicated “Don’t know/Not sure”. The handling of such responses in the data was discussed with PIRg members A.D.-P. and J.J. (John Jackson) It was agreed that for the reporting of frequency distributions the “Don’t know/Not sure” responses would be represented by an additional category.

Analyses were performed using Stata version 15.1 [60].

### 2.6. Qualitative Data Analysis

All focus groups and interviews were voice recorded and transcribed by a GDPR compliant organization. Data were then analyzed using the six-stage framework analysis process outlined in Table 8 [61,62,63]. Framework analysis was a suitable analytical approach as it offers clear, structured steps for summarizing and analyzing qualitative data collected from differing participant groups where broad homogeneity of topics exists. Framework analysis also lends itself to analysis of data where there are multiple researchers involved, as was the case in this study [61].

## 3. Results

### 3.1. Quantitative Findings

#### 3.1.1. Modelling Assumptions

For the multivariable logistic regression analyses reported below there was no evidence of multicollinearity. All but one of the pairwise correlations had an absolute value of less than 0.4, the largest correlation being 0.52. Outliers did not have an influence on the findings. Examination of the other assumptions, including the linearity of the logit links, indicated that multivariable logistic regression was an appropriate approach.

#### 3.1.2. Background Information

Around three quarters of the participating SMEs employed fewer than 50 staff. Approximately 60% were engaged in service activities, 20% in manufacturing, and the remainder were in other sectors. Around 85% had been trading for more than ten years. Fewer than 10% had more than half of their employees on casual contracts and one fifth employed more than half of their staff on part-time contracts. For one third of the SMEs, males represented more than half of the workforce. For two thirds, more than half of the employees undertook manual or routine work. Most (80%) stated that a minority of their employees were from a minority ethnic background.

#### 3.1.3. SME Engagement with Available Workplace Health and Wellbeing Support

Participants were asked to state whether, in the previous 12 months, their SME had made use of the four wellbeing support services available. Around one-sixth (15.5%) of the SMEs had made use of health assessments/NHS checks. For all other types of support, less than 10% of participants reported their SME had used the help available (Table 9). Around 80% of the SMEs had not used any of these sources of support.

The only SME characteristic consistently associated with use of these sources of support was the size of the business. Larger SMEs (50+ employees) were more likely to have taken advantage of WHP (13.3% vs. 1.4%, *p* = 0.001), regional accreditation scheme (6.7% vs. 0.0%, *p* = 0.007), health assessments/NHS checks (23.3% vs. 12.3%, *p* = 0.013) and wellbeing workshops (16.7% vs. 5.5%, *p* = 0.012). SMEs where more than 50% were men were more likely to use the WHP (9.4% vs. 1.5%, *p* = 0.035) and those with predominantly manual employees were more likely to have used wellbeing workshops (14.7% vs. 6.3%, *p* = 0.031).

**Figure 1:**
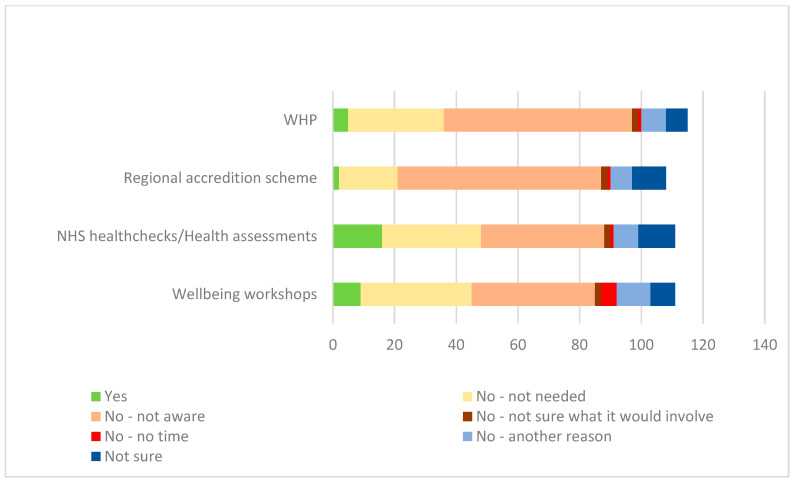
Reasons for not accessing the four key wellbeing support services available locally.

Figure 1 and Table 10 show, for each of the four sources of support, the frequencies of reasons given for not accessing the available support. In Figure 1, the columns extend beyond the total number of participants as more than one reason could be chosen by each participant.

Being unaware of the support available was the predominant reason for not engaging with the WHP and regional accreditation scheme, whereas for health checks/assessments and wellbeing workshops lack of awareness and a perception that the support was not needed were both important.

#### 3.1.4. Workplace Health and Wellbeing Practice and Support Offered via the SME

##### Existence of an Agreed Workplace Health and Wellbeing Strategy or Plan

More than half (58.3%) of SMEs had an agreed workplace health and wellbeing strategy or plan and around one third (30.1%) did not. Some were not sure (11.7%).

Univariate analysis showed that having an agreed workplace health and wellbeing strategy/plan in place was more likely for SMEs with more than half of employees on part-time contracts (85.0% vs. 61.4%, *p* = 0.049), those having a person or department with responsibility for promoting staff health and wellbeing (75.4% vs. 46.9%, *p* = 0.007), and those with a recognized trade union (88.2% vs. 58.8%, *p* = 0.023). It was less likely for SMEs where more than half of employees were men (43.3% vs. 76.3%, *p* = 0.002), and those in either the manufacturing or construction sectors (42.9% vs. 72.9%, *p* = 0.011). There was evidence that SMEs trading for more than 10 years were more likely to have a strategy/plan (69.5% vs. 38.5%, *p* = 0.055).

Multivariable logistic regression performed with adjustment for all the univariate stage variables showed no explanatory variables were clearly associated with the existence of a health and wellbeing strategy/plan (Table 11). Only one outlier was identified, and analysis with this observation removed produced almost identical findings.

Consultation of Employees About Their Health and Wellbeing Needs (e.g., Through a Survey, Needs Assessment or at an Appraisal).

Most (84.5%) SMEs consulted employees about their health and wellbeing needs. A few did not, (13.6%) and only two participants were unsure. Univariate analysis showed that consultation of employees about their health and wellbeing needs was more likely for SMEs with a person or department with specific responsibility for promoting staff health and wellbeing (93.3% vs. 73.7%, *p* = 0.007). Consultation was less likely for SMEs with majority male employees (74.2% vs. 91.2%, *p* = 0.033), and those in either the manufacturing or construction sectors (69.6% vs. 91.0%, *p* = 0.015).

Multivariable logistic regression with adjustment for these variables showed that consultation of employees was more likely if there was a person or department with specific responsibility for promoting staff health and wellbeing (Table 12). Only one outlier was identified and its removal had minimal impact on the findings.

##### Types of Workplace Wellbeing Support Provision Available to Employees via Their SME

**Figure 2:**
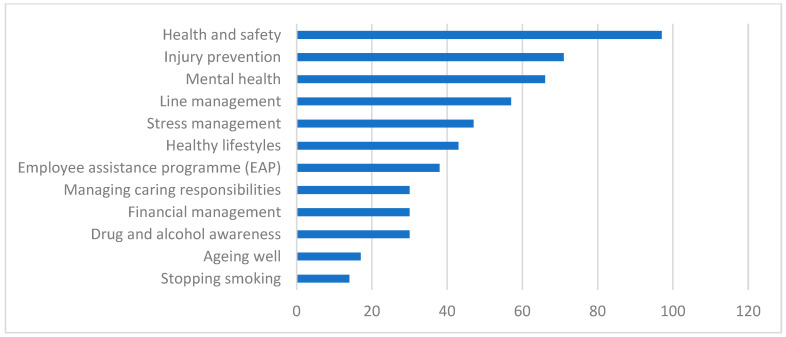
Types of workplace health and wellbeing provision available to employees via their SMEs.

The workplace wellbeing provision available to employees is shown in Figure 2 and Table 13. Types of provision most commonly available were health and safety (94%) and injury prevention (69%). Provision least likely to be available included ageing well (17%) and stopping smoking (14%).

Findings from the multivariable logistic regression analyses on types of support offered are summarized in Table 14. Each analysis was adjusted for the SME characteristics significantly associated at the univariate level with that type of provision, using the process described above, for the existence of a workplace plan and the consultation of employees. This table shows only the characteristics with a significant association for the type of provision concerned.

Where appropriate, the types of provision have been grouped into pairs and clusters to reflect the guidelines for a holistic framework of wellbeing support outlined by CIPD, the UK professional people management body [64]. This was deemed an appropriate framework for grouping provision as it incorporates seven inter-related ‘domains’ of employee wellbeing, to provide a holistic framework reflecting many of the aspects of employer provision that previous research suggests support workplace wellbeing [32,33].

Three SME characteristics were associated with availability of wellbeing provision: having a person with specific responsibility for promoting staff health and wellbeing; having a recognized trade union; having more than half of employees on casual contracts. Having someone with specific responsibility for health and wellbeing was particularly beneficial with around ten times the odds of support for mental health (mental health and wellbeing pairing), and around three to four times the odds for stress management, financial management, caring responsibilities (broader personal wellbeing pairing), and having an employee support programme in place. The presence of a trade union was positively associated with support pertaining to caring responsibilities, having an employee support programme in place and healthy lifestyle support (physical health and wellbeing cluster). SMEs with more than half of employees on casual contracts were associated with a lower likelihood of provision for physical health and safety.

#### 3.1.5. SMEs’ Workplace Health and Wellbeing Support Needs

Reported SME workplace health and wellbeing support needs are shown in Table 15. Each item in the questionnaire was selected by at least one third of participants. Support needs most mentioned were mental health awareness (56%) and NHS health checks (55%).

In the multiple logistic regression analyses, after adjustment for other explanatory variables only SME size, having fewer than 50 employees or not, was significantly associated with any of the areas of support need (Table 16). Participants from smaller SMEs were less likely to state a need for support in the following areas: identifying the health and wellbeing needs of staff, conducting a staff wellbeing survey, and healthy ‘behaviour change’ support.

#### 3.1.6. SMEs’ Views and Attitudes About Workplace Health and Wellbeing

Participants were asked to respond to the statement: “Employers have a responsibility to support the health and wellbeing of employees” using one of the options “Strongly agree” (scored as 5), “Agree” (4), “Neither agree nor disagree” (3), “Disagree” (2), “Strongly disagree” (1). Of the 103 participants, 59 (57.3%) strongly agreed, 40 (38.8%) agreed and 4 (3.9%) neither agreed nor disagreed. No participant disagreed with this statement. The only SME characteristic associated with these scores was having a recognized trade union, for which a response of “Strongly agree” was more likely (83.3% vs. 52.0%, *p* = 0.016).

### 3.2. Qualitative Findings

Since the study aimed to explore SME engagement with available workplace health and wellbeing support, provision of support to staff, and staff uptake of employer-provided support, the combined qualitative data from the three participant groups is organized in themes under these categories. Data from each participant group contributed to at least two categories, and our analysis across participants allowed for the identification of common themes.

Various themes and sub-themes related to ‘SME engagement with workplace health and wellbeing (WHW) support’, ‘SME provision for employees’, and ‘employee uptake of employer-provided support’ were developed during the mapping and interpretation stages of the framework analysis process. These are described below alongside illustrative quotes. Table 17 provides an overview of the themes and sub-themes.

## 4. SME Engagement with Workplace Health and Wellbeing Support

### 4.1. Knowledge and Awareness of the Workplace Health and Wellbeing ‘Offer’

This theme relates to SME knowledge and awareness of the existence and nature of available workplace wellbeing support for their organization. Two sub-themes were identified.

#### 4.1.1. Overall Level of Awareness of Available Support

While some SMEs were aware of or had accessed local authority support for their organization, participants highlighted low levels of awareness of available support, stating this was a key barrier to SME engagement.

“*We haven’t had no interaction with any councils, or anything about any health and wellbeing within the workplace. So literally, it would just be myself googling and going through Citizens’ Advice, going through the government webpages to try and find resolutions if they were needed.*”

(SME interview 7)

SMEs identified a lack of communication from the local authority regarding health and wellbeing support for their SME, and limited publicity around the topic. They and stakeholders agreed that SMEs lacked knowledge about what was available and how to access it.

#### 4.1.2. Clarity and Accessibility of Messaging and Communications

Participants agreed that communication and messaging from the local authority about available workplace wellbeing support required improvement if SMEs were to be more effectively engaged. They highlighted the importance of clear, accessible messaging, written in meaningful, business-friendly language, understandable across sectors. Participants emphasized the importance of using a range of communication modes, including social media, emails, in-person events, and mailshots. Including reference to the business benefits of workplace wellbeing was highlighted as likely to encourage engagement.

“*I think it’s, a lot of it I’ve found in this sphere can be to do with the language we use as well…we didn’t talk enough about growth, productivity, retention, recruitment, we perhaps didn’t use the language that would entice a business to better understand…business friendly language isn’t it, so sometimes we don’t perhaps… the offer is brilliant, what we do is great, but we just don’t get the definition or the language right.*”

(Stakeholder focus group, participant 8)

### 4.2. Organizational Perceptions and Conceptualizations

This theme focuses on SMEs’ perceptions and conceptualizations of workplace health and wellbeing support. It includes SMEs’ understandings of what workplace health and wellbeing support might consist of, and its relevance to their organizations. Four sub-themes were identified.

#### 4.2.1. Conceptualization of Workplace Health and Wellbeing

Stakeholder and SME participants held varying conceptualizations of workplace health and wellbeing. There was understanding that the topic related to the health and wellbeing of employees, but variation in what topics and issues were included. Aspects of workplace health and wellbeing mentioned included physical and mental wellbeing, home and family life, and support for personal issues that impact wellbeing (such as debt or bereavement). A distinction between understanding ‘workplace wellbeing’ primarily in terms of health and safety at work and conceptualizing it as broader employee wellbeing was also identified.

Also apparent in the perspectives of some SMEs was the centrality of the workplace cultural environment to supporting employee wellbeing:

“*…to me it’s [workplace wellbeing] making sure people feel, within the workplace, that there is support there, that they know who to go to, they know that they’re not going to be penalized or labelled or anything like that. I think wellbeing covers such a huge aspect and there’s not many people that would actually vocalize, ‘I’m struggling financially, I’m struggling with my mental health’, and it’s about making sure that it’s not a taboo subject and things like that.*”

(SME interview 8)

SMEs also discussed the impact of the COVID-19 pandemic on the priority given to workplace wellbeing, their understanding of the topic, and the support that they deemed appropriate for their staff. Wellbeing issues had gained increased visibility since the pandemic and for some SMEs there was a heightened appreciation of the importance of including mental wellbeing in conceptualizations of workplace wellbeing.

“*The main focus came about as a result of Covid. The business has focused on health and wellbeing more over the last 2 years.*”

(SME ‘other comments’ questionnaire response)

#### 4.2.2. Perception of Organizational Burden

SMEs perceived engagement with workplace wellbeing support as involving additional burden for their organization. Typically, this was in terms of financial outlay or additional time or effort. Participants suggested that this perception was a significant barrier to SME engagement with support, particularly apparent for SMEs with little experience of accessing external support.

Participants also discussed a range of practical barriers to engagement with and provision of support, including time and financial constraints, and these are discussed in later sections of this paper. However, it was clear that initial perception of burden was itself a barrier to SME uptake of support.

“*…also a lot of companies don’t think that they’ve got the time…I don’t know, they’ve got a fear that it’s going to cost them a lot of money, I’m not sure why but that’s what I found out anyway.*”

(Stakeholder focus group, participant 4)

Our analysis suggests that a lack of SME knowledge about the precise nature and implications of engaging with support enabled such perceptions to persist. Participants discussed the importance of clarifying with SMEs that support could be free, light-touch, and involve little or no additional burden.

“*For me the easiest kind of sell to businesses is always something that’s free and something they haven’t got to put any work into.*”

(Stakeholder focus group, participant 1)

#### 4.2.3. Perception of Organizational Need

This sub-theme relates to SMEs’ perceptions of the needs of their organization and their degree of recognition that it might benefit from external support. Some SME participants highlighted the importance of gauging the wellbeing needs of their staff to ensure that they were met. However, some stated they had not accessed support as their employees had few, if any, wellbeing needs, and therefore accessing external support and providing support for employees was unnecessary. Importantly, those who cited a lack of employee need as the main reason for their lack of provision, did not conduct rigorous, routine assessments of employee needs, so may have been underestimating the degree of support required.

“*I genuinely don’t feel that anything like [workplace wellbeing support services]… that has ever been called for, or needed…If there’s something needed, it would be something I’d have to address, and I would find a way of looking at it. I don’t feel I have actually needed anything like that, or felt it would benefit the company in any way.*”

(SME interview 6)

#### 4.2.4. Fear of Consequences

Stakeholders stated that SMEs were sometimes resistant to engaging with external support because of the perception that engaging might have ‘negative’ consequences for their organization, for example, enforcement action. SMEs were also said to sometimes be resistant due to fear that accessing support might result in the highlighting of wellbeing issues that required costly intervention.

“*…their businesses might feel a bit, as she said a bit afraid to have the public health team in just in case they find something that, you know, oh is there something that they’re not doing or is there something that they’re not doing right?*”

(Stakeholder focus group, participant 2)

### 4.3. The Importance of Flexibility

Flexibility was a common theme, both in terms of the support on offer for SMEs and in relation to encouraging SME uptake. Two sub-themes were identified.

#### 4.3.1. The Need for a Varied and Multi-Pronged Approach

Stakeholders highlighted the need to apply a range of approaches to maximize SME engagement with support. They emphasized SMEs’ different needs and resources, and the differing ways SMEs engage with marketing materials, agreeing that a ‘one size fits all’ approach was inappropriate and that simultaneously employing multiple engagement approaches was most effective.

“*…so I think it’s how do we get that message out to the employers based on the team and there’s so much to offer, so it’s quite wide again, isn’t it…we’ve got to use different platforms to do it…*”

(Stakeholder focus group, participant 3)

“*Then there’s what really resonates with some of our businesses, and we always talk about proactively engaging the disengaged businesses, those hard to reach businesses, those businesses that believe it or not don’t have computers, they don’t have Twitter, they wouldn’t have an email address to sign up to a newsletter, they absolutely do exist in [the area].*”

(Stakeholder focus group, participant 8)

#### 4.3.2. Tailoring Support to SME Needs

Stakeholders also discussed the importance to SME engagement of having a flexible range of support available and adapting the support offered, or how it is delivered, to meet the differing needs and preferences of SMEs. For them, responsiveness to the needs of SMEs was vital, and tailoring support helped to encourage engagement.

“*…so it’s that element of making our services tailored so that they have that accessibility, that they do feel that although I am a small business and I don’t have a computer I can still engage in this stuff…*”

(Stakeholder focus group, participant 6)

### 4.4. The Importance of Partnerships and Relationships

Participants discussed the importance of effective relationships and partnership working among those attempting to encourage SME uptake of support, and between SMEs and those offering support. Two sub-themes were identified.

#### 4.4.1. Joint and Collaborative Working

Stakeholders recognized that workplace health and wellbeing was a priority for various council and health teams. They therefore felt that encouraging SME take-up of support was best achieved through joint and collaborative working between those with converging wellbeing remits, including those working in areas as diverse as corporate social responsibility, health and safety, and public health. Participants stated that coordination between multiple teams, in terms of strategy and messaging around wellbeing support, was needed to optimize SME uptake.

“*Yeah, yeah, so I think my two big things would be collaboration, so I don’t think there’s anything we do as a [team name] team that can be done in isolation really, most of the time you need to bring a partner in or a colleague, or another organization, so collaboration would be key.*”

(Stakeholder focus group, participant 8)

#### 4.4.2. Building Trusted Relationships Between SMEs and Those Offering Support

Where SMEs did access support, contact was usually via a single person within the SME. For stakeholders, accessing this key person within the SME was vital to encouraging the SME to access support. However, making contact with them was often challenging.

“*…it’s not about the offer because, you know, the workplace health offer is a good one, I think whatever it looks like it’s, if it’s got the right person selling it to the right person to hear it there’s always something good that the business can take from it whatever the offer is, it’s about being in front of the right person and that’s the difficult bit I think.*”

(Stakeholder, focus group, participant 1)

Participants highlighted the importance of a trusting relationship between those offering support and the person within the SME in breaking down barriers to engagement.

“*So I think it’s linking in with them [local authority], making sure that we’re aware of them, you know, if they’ve got services that are running that our staff might want to get involved in, whether it’s through work or outside of work, and things like that, really…So I think it’s just one of those, really, to just sort of start building up that networking, that relationship.*”

(SME interview 8)

## 5. SME Provision for Employees (Four Themes)

### 5.1. Sufficiency of Provision

This first theme related to SME workplace health and wellbeing provision for employees, focuses on SME and employee participants’ discussions about the extent of support provided, and views on its sufficiency in meeting employee needs. Two sub-themes were identified.

#### 5.1.1. Varied Levels of Provision

The extent and type of provision for employees varied between SMEs. Types of provision available through SME employers involved in this study included social events, wellbeing groups, NHS health check provision, and ad hoc support for health and wellbeing issues. Some SMEs provided multiple types of wellbeing support for their employees while others offered very limited provision.

“*And in the three years I’ve been here, that’s the first time I’ve known somebody to come in and do a health check, and saying about your diet and not to smoke, and whatever else. Other than that, I’ve never really known my company to offer any healthcare in any sort of way, to be honest…*”

(Employee interview 2)

#### 5.1.2. Providing a Supportive Environment

A common theme among SME participants was the desire to provide a working environment conducive to employee wellbeing. This tended to be the case even where routine workplace wellbeing provision was limited. SMEs stated their general commitment to staff wellbeing and emphasized their desire to ensure that, even if direct provision was limited, their staff felt valued and were able to gain support for wellbeing issues. This desire to provide a generally supportive environment for employees was central to most SMEs’ understanding of workplace health and wellbeing.

“*So looking at what we can put in place that, you know, at the end of the day you can’t always pay the highest salary, but they would like to make sure that employees feel valued and the wellbeing is there, really, within the organization.*”

(SME interview 8)

Some employees also stated that even where routine workplace wellbeing provision was limited—for example, where lack of organizational resources hindered provision—they felt that their employer cared about their wellbeing and did their best to support them in the workplace.

### 5.2. Matching Provision to Employee Needs

A common theme in SME interviews was the extent to which SMEs ensure that workplace wellbeing provision meets employee needs and how they accomplish this. Three sub-themes were identified.

#### 5.2.1. Informality of Staff Health and Wellbeing Needs Assessment

SMEs did not tend to conduct formal, periodic, wellbeing needs surveys or consultations. Instead, information about wellbeing need was typically gathered from individual employees on an ad hoc basis via informal conversations and discussions. Examples included discussions between employees and managers when an employee had a specific wellbeing concern, informal talks with employees to gauge general wellbeing, and brief wellbeing discussions during annual appraisals.

“*There’s some sort of informal discussions, or just generally, you’ll just go around the workshop and you’ll just be like, are you okay sort of thing? So we don’t do it formally…*”

(SME interview 7)

“*We have our appraisals as well, where we speak to staff individually, and part of that appraisal is, ‘how are you feeling?’. It’s very informal, actually, and we give people an opportunity, again, we have a blank page on the appraisal document.*”

(SME interview 3)

#### 5.2.2. Variation in Awareness of Staff Wellbeing Needs

Variable levels of SME awareness of staff wellbeing needs were common. Where SMEs lacked awareness of employees’ needs, the lack of systematic needs assessment highlighted above appeared to be a contributory factor. While some employee participants stated that their employer did attempt to gauge their needs, others described their employers’ apparent lack of interest in their health and wellbeing, and lack of awareness of their requirements.

“*… but health and wellbeing really no, they don’t, like they don’t really ask, they don’t really like check up on our health or anything or I don’t know, do anything extra like that, no.*”

(Employee Interview 6)

Some participants suggested that factors such as organizational size and the location and mode of employee work influenced the ability of SMEs to assess employee need. For example, some stated that having fewer employees might facilitate a greater degree of manager-employee interaction, and higher levels of organizational awareness of employee needs. Participants also suggested that factors such as remote or off-site working hindered the ability of SMEs to gauge the needs of employees.

“*It’s harder to spot [wellbeing needs] when you’re in a bigger organization, if you’re a manufacturer or you’ve got a big warehouse, you don’t always know what the staff are doing.*”

(Stakeholder focus group, participant 3)

“*Because we are not a massive team, I have enough time to speak to individuals within the business.*”

(SME ‘other comments’ questionnaire response)

#### 5.2.3. Reactive Rather Than Proactive Approach

SMEs tended to adopt a ‘reactive’ rather than ‘proactive’ approach to workplace health and wellbeing provision. There were some examples provided of proactive, preventative workplace health and wellbeing practice within SMEs. However, employee wellbeing needs were typically addressed after a wellbeing issue had arisen, rather than routine needs assessment resulting in workplace wellbeing issues being pre-empted or prevented.

“*I’d say that most of the organizations probably are reactive, they’ll identify an issue and then they’ll want to do something about it, probably with an employee who’s been ill a while because they’ve had, you know, some kind of mental health issue or accident and then they think okay, and it could be based on health, health and safety issues that have been identified and they’ve decided they need to do something,*”

(Stakeholder focus group participant 3)

“*If somebody came and said, oh, I need this, or I need that, I probably would think, oh, yeah, perhaps they need the health check…but, yeah, I’ve probably not highlighted it.*”

(SME interview 2)

Stakeholders highlighted the importance to effective workplace health and wellbeing, of proactive approaches that identify and address staff needs at an early stage.

### 5.3. Drivers of SME Provision

Data from all participants identified four themes that explain SMEs’ rationales for and drivers of workplace wellbeing provision. In some cases, SMEs identified multiple motivations for provision.

#### 5.3.1. Business Case and the Importance of Staff to Business Performance

The ‘business case’ was a major motivator for SMEs’ workplace wellbeing provision. The business case in this context included recognition that employee wellbeing is important for staff retention, business productivity, reduced absenteeism, or profitability and sustainability. SME participants were typically aware of the potential for employee health and wellbeing to affect commercial performance, and our analysis suggests this was an important motivator for provision.

“*And I think that’s also what drives it, really, because at the end of the day, if we’re doing more wellbeing for the members of staff and, you know, they’re happy and, you know, they feel valued and that the wellbeing is there, then I think productivity is better as well.*”

(SME interview 8)

#### 5.3.2. Organizational Ethos and Culture

Participants highlighted the importance of a wellbeing ethos and culture within the SME in underpinning and motivating the provision of support for staff. Those SMEs who did express a strong commitment to workplace wellbeing, typically stated that looking after their employees’ wellbeing reflected the caring and pastoral culture of their organization.

“*So I think it is part of just the ethos of the organization, as a charity, that’s what we do for people. So it’s quite natural for us, for that to just sit in our whole being, if you know what I mean? That’s what we do.*”

(SME interview 3)

#### 5.3.3. Compliance

Compliance with legislation and statutory requirements was also a common driver of workplace wellbeing support. Compliance was primarily discussed in terms of adherence to workplace health and safety legislation. Participants discussed the motivating role of legal requirements.

“*Because you know how legislation and things change every day, every week, every month, it would be good to keep on top of it, just to make sure that we’re compliant and we’re making sure that we’ve safeguar-… Well, I’m saying safeguarding, we’re not really a [type of business removed] or anything, but just making sure that people are safe whilst they’re at work, and outside of work.*”

(SME interview 5)

“*If the government isn’t putting legislation to provide a work-life balance then employers won’t see it as a necessity…So until employee health and wellbeing is seen as a priority by the government then we’re literally going to be depending on the, out of the kindness of employers’ hearts to access certain services.*”

(Employee interview 7)

#### 5.3.4. Importance of Organizational Workplace Wellbeing ‘Champions’

Our analysis also highlighted the importance of workplace wellbeing ‘champions’ within the SME to the provision of support. These individuals were instrumental in establishing the SME’s ethos and practical approach to workplace wellbeing or developing, coordinating, or promoting the provision of employee support. Champions included business owners, members of SMEs’ senior management teams, human resource managers, and other staff members with responsibility for coordinating employee wellbeing provision. They were commonly pivotal in shaping the organization’s approach and the extent of provision. Senior management commitment to staff workplace wellbeing was a particularly important driver.

“*I think it drives, really, from the MD, I think, you know, he wants to have, I suppose, a happy workforce, a good workforce…*”

(SME interviewee 8)

Conversely, the lack of a champion with time to devote to coordinating workplace wellbeing provision was highlighted as a barrier to provision. This was a particular issue for SMEs with limited financial and staff resources to devote to wellbeing provision. One SME representative explained:

“*I suppose, because it would be me that would have to do it, and I’m juggling a lot of other things…*”

(SME interview 1)

### 5.4. Practical Issues

Various practical issues were identified in the data as hampering the ability of SMEs to provide workplace wellbeing support. Two sub-themes were identified.

#### 5.4.1. Prioritization—Balancing Wellbeing Provision and Business Sustainability

Balancing workplace health and wellbeing provision with the demands of running an organization and ensuring sustainability was highlighted as a concern for small and larger SMEs, and those in the private and not-for-profit sectors. It manifested as either a general de-prioritization of workplace wellbeing support in order to focus on main business operations or limited de-prioritization at certain points in the annual business cycle. Both resulted in reduced focus on staff wellbeing. In addition, our data were collected following the COVID-19 pandemic when the economic environment remained challenging for business, with rising energy prices and high inflation. These cost pressures may have influenced SMEs perspectives on workplace wellbeing and the provision of support for staff.

“*I’ll be having discussions with the MD, and I think it’s just that balance, like they’re very keen on publicizing wellbeing and that, but obviously we do have to be aware that we’ve also got to run the service, so it’s getting a balance, and obviously costs and things like that.*”

(SME interview 8)

“*It’s very important. I just think in the heat of the moment, when it’s very busy and they’re focusing on business and profit, and cash flow it possibly can get overlooked. Not overlooked, but put to one side. And it doesn’t make it any less important, and it gets pulled back to the forefront again, but it can be. It’s very easy to do that, isn’t it?*”

(SME interview 1)

#### 5.4.2. Lack of Organizational Resources

A lack of organizational resources to support workplace wellbeing was highlighted across participant groups as a practical issue that hampered provision for staff. This included a lack of staffing to develop and coordinate wellbeing provision, limited financial resources, limited wellbeing knowledge and expertise, and a lack of wellbeing related policies and processes.

“*Had there been like a person that we can directly talk to and you know that she’s in charge of this specific like our wellbeing, our stuff, then I think it would’ve been easier, like it would’ve been like better because that’s literally her job to listen to us and to advise us and to do this. But obviously really that’s, no-one really has that role and everyone just goes to my manager and she literally, she manages everything…and she’s just too busy with so much of the stuff that she’s running.*”

(Staff interview 6)

## 6. Employee Uptake of Employer-Provided Support

Five themes were developed that illuminate our understanding of SME employee uptake of employer-provided workplace wellbeing support.

### 6.1. Lack of Awareness of the Range of Employer-Provided Support

Where workplace wellbeing provision was available, employees sometimes lacked knowledge of what was on offer. This was most apparent in interviews with employees who worked away from their employer’s main premises, some of whom were completely unaware of support available. Even staff working on-site, were sometimes aware of only a small proportion of the available provision.

“*As far as I’m aware, it’s pretty much the same across the board. I haven’t spoken to any other [role removed] who have said to me, oh, no, they’ve got this or that going on. They’ve never really—I’ve never heard about any sort of schemes or plans that my employers offer.*”

(SME staff interview 2)

### 6.2. Practical Issues

As with the provision of workplace wellbeing support by SME employers, practical issues were identified that hampered employee uptake of workplace wellbeing support. Two sub-themes were identified.

#### 6.2.1. Logistical Difficulties—Employee Working Location or Role

Staff work role or location were sometimes a barrier to uptake of workplace wellbeing provision. Enabling access to provision might, for example, require removing employees from productive work for a time, causing operational disruption. These issues were especially apparent where staff worked in manual manufacturing roles or were often off-site (such as driving and delivery roles).

“*But the problem we’ve got is, the time, and it’s releasing everybody because everyone’s here and it’s fast-paced, and you’ve got to get an order through the door.*”

(SME interview 5)

“*It’s very important to us [workplace wellbeing provision], but it’s very cost-prohibitive…if I have to bring in an occupational health specialist, okay, that could cost me anywhere between [costs removed] for a van to arrive on-site, but I’ve got to get the workers back to be seen, and I’ve also got to pay them…So it’s an absolute double whammy for us because we’re paying for, obviously, their care, but we’re also having to pay their wages. And we’ve also got the lost revenue, so it’s a very fine line to balance.*”

(SME interview 4)

#### 6.2.2. The Need to Timetable Workplace Wellbeing Support Within Working Hours

SMEs and staff commonly highlighted the difficulty of fitting staff engagement with workplace health and wellbeing provision into employees’ working patterns, but also the importance of trying to do this in their working day.

“*People just have busy lives, so as long as you do it [engagement with workplace wellbeing support] within worktime and it doesn’t take them out of their worktime, and they’re getting paid for doing it in worktime, then that’s fine. It’s if it takes it—if it happens any other times, as long as you can include it as part of their job on that day…*”

(SME interview 1)

Expecting employees to participate outside of paid working time was regarded as unfeasible, as staff were reluctant to take part in workplace wellbeing provision outside of working hours. This was challenging for SMEs keen to encourage employee wellbeing provision uptake.

“*It’s the time, and that’s the only thing. We are so fast-paced here, and with people on a national minimum wage, nobody can afford to stay back. And in the current climate, it’s not fair to keep people back and expecting them to stay back.*”

(SME interview 5)

### 6.3. Employees’ Reluctance to Disclose Issues

SMEs and employees stated that employee reluctance to disclose their wellbeing support needs to their employer hampers awareness of staff wellbeing requirements, reducing the likelihood that employers can provide appropriate support. The reasons for this reluctance are reflected in the three sub-themes below.

#### 6.3.1. General Reluctance to Disclose

A general reluctance from staff to discuss health and wellbeing issues was identified and attributed to preferring to keep health and wellbeing issues private or manage them independently. This general reluctance was said to be particularly apparent among certain groups of workers, such as older workers and men.

“*So I just feel that for me, personally, I think it [wellbeing] is important. However, getting that message across to people, it’s not as easy as you think it is, because some people don’t want to talk about it.*”

(SME interview 5)

“*…don’t know if it’s because, obviously, men feel that they need to be more proud, that they don’t talk about it, sort of thing…we’re not a younger generation, and the majority of our gents here, there’s more of an older generation and it’s quite hard to break that habit.*”

(SME interview 7)

#### 6.3.2. Concerns About Confidentiality

Concerns about the confidentiality of information they might disclose was highlighted as a barrier to employees sharing their wellbeing needs with employers. These concerns were described as a particular barrier in smaller organizations, where employees might be more fearful of privacy breaches.

“*…the thing is, I feel that people want to talk, but don’t want to talk because if they do open up, just in case it gets leaked, confidentiality is very key here.*”

(SME interview 5)

Participants discussed the need for discreet and anonymous routes for staff to disclose their wellbeing needs and access support.

#### 6.3.3. Suspicions Regarding Employer Motivations and Fear of Negative Consequences

A small number of participants also suggested that employee suspicion about the motives of employers’ who request information about health and wellbeing needs, might be a barrier to disclosure. These participants suggested that a lack of employer-employee trust, may lead to employees fearing unfavourable treatment should their employer become aware of a health or wellbeing issue.

“*I think if anybody doesn’t take up the opportunity, it’s all about trust…Thinking that there may be a hidden agenda why we’ve made this available, or we’re getting feedback that can be used for ulterior motives.*”

(Employee interview 1)

### 6.4. Organizational Culture and Approach/Environment

Our analysis suggests that the workplace wellbeing culture of SMEs may affect the likelihood that employees will take up support. There were two sub-themes.

#### 6.4.1. Employer Focus and Prioritization

Participants suggested that top-down organizational prioritization and promotion of health and wellbeing help to create an open environment around the topic, enabling identification of health and wellbeing needs, and provision and uptake of support.

“*…it’s a tough one trying to get anyone involved in anything. I think it’s just us driving it from the top, really. We drive it from the top and we’re enthusiastic, and it usually filters down, and that is usually how things work…And you do have to drive it, then they’ll [employees] take it onboard and get involved.*”

(SME interview 1)

Our data included accounts of SMEs that prioritized wellbeing and those that did not. Some employees felt that their employer was not interested in their wellbeing, which was reflected in little, if any, workplace wellbeing provision or routine discussion of wellbeing issues. For others, although routine health and wellbeing provision was limited, they still felt there was an organizational interest in workplace wellbeing and that their employer would offer what support they could where a need was identified.

“*She doesn’t just listen to you and then just ignore it or put it aside, she actually makes sure that things have been put in place to help you…*”

(Employee interview 5)

#### 6.4.2. The Importance of an ‘Open’ Health and Wellbeing Culture

Similarly, participants discussed the importance of employee uptake of support, of an ‘open’ culture that welcomes discussion of workplace wellbeing issues. Examples of this include ensuring that discussing health and wellbeing issues is normalized and routine.

“*And one of our big focuses at the moment, is on LGBTQ provision…and trying to create a cultural shift, really. And, again, resistance among certain cultural elements of our [organizational] community, towards a much more liberal outlook. And, again, that’s about listening to people and getting people to feel confident that they can access wellbeing and mental health services, regardless of gender, sexuality, religious standpoint. So there’s things like that where there are clear barriers and we are trying to combat those barriers.*”

(Employee interview 3)

### 6.5. Staff Workplace Health and Wellbeing Beliefs and Attitudes

Finally, data from SMEs and staff highlights the impact of employees’ workplace health and wellbeing beliefs, attitudes and conceptualizations, on their engagement with provision. Two sub-themes were identified.

#### Employees’ Reactive/Remedial Approach to Workplace Health and Wellbeing

Employees tended to view access to workplace health and wellbeing support, as something to access where there was a specific need, rather than to prevent issues occurring. This was apparent in employees’ discussions of why they had not accessed available provision, with a common reason being that they did not have any current wellbeing issues that required support. Their responses may, of course, reflect employers’ conceptualizations or the types of provision publicized and made available, which may have been predominantly reactive rather than preventative.

“*Yeah. I wouldn’t say I’ve used it specifically because I haven’t really had any problems but I know that it’s there for me, kind of thing. If I like need to look for it, it’s always there for me. So if I did have a problem, it’s easy just to like talk to someone really and then they can help me.*”

(Employee interview 4)

“*I think the reason I’m not really accessing at the moment is there’s nothing personally that I really need at the moment, if I did need something then I would, obviously I would access the services that are offered…*”

(Employee interview 8)

### 6.6. Beliefs About the Appropriateness of Discussing Wellbeing Issues

Some employees held views about the appropriateness of discussing workplace wellbeing issues that acted as barriers to engagement with employer provision. For some, this was the belief that wellbeing issues were personal and therefore did not belong in the workplace. For others, the view that workplace wellbeing is ‘touchy feely’, concerned with the expression of emotion, was said to act as a barrier to engagement.

“*And the wellbeing sometimes feels like a bit of a tag-on, or a bit of a liberal, lefty kind of think point that not everybody wants or needs…people have said, ‘I don’t need my workplace to provide mental health and wellbeing services, I have a strong family unit, I have a strong friendship unit, I don’t need that to be provided by my workplace. I come to work, to work, and my personal life, is my personal life.’*”

(Employee interview 3)

Some participants highlighted variations in views and beliefs about workplace wellbeing held by those with different cultural, religious, ethnic, gender and age characteristics, stating that in some cases, attitudes and perspectives towards workplace wellbeing reflected social, cultural, and demographic factors.

## 7. Discussion

**Figure 3:**
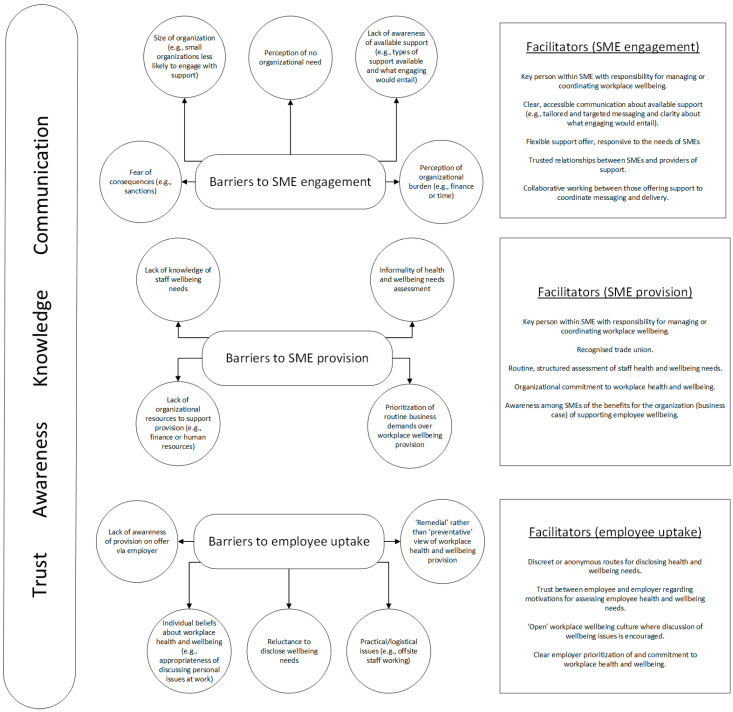
Barriers and facilitators to SME engagement with workplace wellbeing support, SME workplace wellbeing provision for employees, and employee uptake of employer-provided provision.

Our quantitative analysis of SME survey data and the range of themes developed through our qualitative analysis, enabled us to identify various factors that act as barriers to or facilitators of the three aspects of SME workplace health and wellbeing provision and uptake that were the focus of this study: SME engagement with local authority support, SME wellbeing provision for employees, and employee uptake of provision. Figure 3 provides a visual overview of the barriers and facilitators identified through our analyses.

Our qualitative analysis highlighted the inter-related nature of the identified barriers. For example, for the ‘SME provision for employees’ aspect, the barrier of ‘lack of knowledge of staff wellbeing needs’, although distinct from ‘informality of health and wellbeing needs assessment’, is also closely related to it, and in some cases, may result from it. Similarly, for the ‘SME engagement with external support’ aspect, ‘lack of awareness of available support’ is a potential contributor to the barrier of SMEs’ ‘perception of organizational burden’. Of course, a range of other factors also potentially contribute to the development of these perceptions.

Our qualitative analysis also suggests that single facilitators may assist in overcoming one or more of the identified barriers. For example, for the ‘SME engagement with external support’ aspect, ‘clear, accessible communication of available support’ may help overcome multiple barriers, such as ‘lack of awareness of available support’, ‘perception of organizational burden’, and ‘fear of consequences’. In addition, as Figure 3 illustrates, we identified four components that our qualitative analysis suggests are important prerequisites of optimal provision and engagement between a local authority or other provider, SMEs and their employees. These are:

- trust (for example between those providing external support for SMEs and the SMEs themselves, and between employers and their employees);
- awareness (for example, awareness of the existence of support for SMEs or provision for employees);
- knowledge (for example, of what engagement with external support will entail or, for employees, the potential benefits of ‘preventative’ engagement with workplace wellbeing support);
- and communication (for example, use of appropriate, tailored communication channels to inform SMEs about support, or SME to employee communication about available provision).

Our use of mixed methods allowed for both breadth and depth of understanding and analysis. In addition to enabling us to identify barriers and facilitators, the thematic analysis provided valuable explanatory benefits, allowing an insight into how the various organizational characteristics, circumstances, barriers and facilitators interlink. To understand the barriers and facilitators identified in this study, it is important that they are viewed as a range of inter-related components rather than as individual, isolated factors.

Our findings suggest that despite a generally positive attitude to workplace health and wellbeing, as evidenced by SMEs’ responses to our attitude survey question, barriers exist to SMEs’ implementation of wellbeing related practices. These findings align with those of previous UK employer surveys [65]. Our findings regarding barriers to SME engagement with external support and provision for employees are also consistent with previous research. For example, studies have identified organizational size [42,66] and lack of knowledge about the availability of suitable support [67,68] as important barriers to organizational engagement with wellbeing support.

An area where our study extends knowledge is in the identification of additional barriers to SME engagement with support such as perception of organizational burden and fear of consequences. Importantly, perceptions of organizational burden may exist independent of the realities of engaging with or providing wellbeing support and provision. For example, engaging with external wellbeing support may have potential resource implications, but our findings suggest that even where free support is available and actual burden is limited, perceptions may persist. For those seeking to encourage greater SME engagement with external wellbeing support, there will likely be benefit from a focus on addressing any SME misconceptions about burden.

Regarding the support needs of SMEs, those with fewer than 50 employees were less likely to state that they required certain types of external support. The reasons for this are unclear, and while on the face of it this finding may seem counterintuitive, our qualitative analysis offers possible explanations. For example, the finding may relate to the perception in smaller organizations that there is no organizational need or a lack of prioritization of wellbeing issues. Further research in this area would help clarify this.

For barriers to organizational workplace wellbeing provision, many of our findings are consistent with extant research. For instance, a lack of organizational resources, including financial resources and organizational structures was highlighted as a barrier in our study and has been highlighted by various studies as a barrier to workplace wellbeing provision to employees [65,69,70], as have lack of awareness of staff wellbeing needs [71] and lack of prioritization of workplace health and wellbeing because of pre-occupation with routine business operations [69,72]. In addition, previous research has found that small business employers may take a reactive rather than preventative approach to occupational health and wellbeing [73]. This too was reflected in our thematic analysis.

Benning et al.’s recent study into the determinants for implementation of preventative workplace health and wellbeing (mental and musculoskeletal) measures in SMEs highlighted various factors that are broadly consistent with the findings of the current study, and some of which demonstrate fit with the four overarching, pre-requisite components identified through our analysis [71]. The study identified the importance to workplace wellbeing, of available resources, awareness and knowledge around workplace health and wellbeing issues, practical issues such as time constraints, staff and SME commitment, communication, trust, and organizational culture and approach. Considering this and other research reporting similar findings (e.g., Spence, 2015 [74]), our analysis suggests that the overarching components of trust, awareness, knowledge and communication around workplace wellbeing may provide fruitful areas of focus for local authorities and SMEs attempting to improve engagement with wellbeing interventions.

Another important addition to knowledge from the current study is our finding about the typical informality of health and wellbeing needs assessments within SMEs, which appears to contribute to the lack of knowledge about staff wellbeing needs and to the perception that there are no significant wellbeing needs within the organization. Although our survey findings indicated that most SMEs consulted staff about their wellbeing needs, the framing of our survey question meant that affirmative answers will have included informal consultation, such as those that occur during annual appraisals. Our qualitative analysis suggested that rigorous, routine, systematic needs assessment was uncommon, something that previous research has highlighted may be important to the effective implementation of health and wellbeing interventions [75,76]. Those wishing to encourage more effective SME wellbeing practice, may find it useful to devote energies to supporting SMEs to routinely and systematically assess staff wellbeing needs.

Our findings about barriers to employee engagement with wellbeing provision also echo those of previous studies that have identified barriers such as staff location and finding time to fit participation around work [71,74] and staff concerns about confidentiality and stigma [72]. Employees’ reactive approaches towards engagement with wellbeing provision, however, is a relatively novel finding, in that previous studies have not directly identified it in the SME context. It may warrant further exploration in future studies, as previous research suggests that employees’ beliefs around wellbeing can impact their engagement with workplace health interventions [74].

Our survey analysis also found evidence of the positive impact on SME workplace wellbeing practice of internal staff resources to coordinate wellbeing provision. For example, consultation with employees and provision of some types of support, notably mental health support, were more likely where there was a person or department with specific responsibility for promoting staff health and wellbeing. This underlines the importance of champions with responsibility for coordinating workplace wellbeing practice and aligns with previous research findings [69,77]. Previous studies have also emphasized the importance of organizational leadership to workplace approaches to wellbeing provision [78,79], and our study similarly found this to be a facilitator of both engagement with support and provision for employees. Similarly, our findings around the need for clear workplace wellbeing communication and messaging reflects those of previous studies [27,75,79].

Our quantitative analysis also indicated that staff resources were associated with wellbeing provision, as were having a recognized trade union, and having more than 50% of employees on casual contracts. The finding about trade union recognition represents another potentially important addition to knowledge, as the existence of trade union recognition was also found to be the only SME characteristic associated with our measure of attitude to workplace health and wellbeing. This suggests that, as well as being more likely to provide certain types of wellbeing provision, SMEs with a recognized trade union may also have a greater sense of responsibility for supporting the health and wellbeing of employees. We cannot be certain from our analysis, about the reasons or mechanisms underlying the relationship between union recognition and employers’ attitude to workplace wellbeing and provision for workers. There is also a lack of previous research into this relationship, the exceptions being the topic of health and safety, which has been extensively researched in relation to trade union influence on workplace safety implementation [79]. There is also little research into the relationship between union recognition and broader employee wellbeing, although a small number of recent studies have suggested a positive relationship between union recognition and worker health and wellbeing [80,81]. More research on this topic is needed to understand the relationship between union recognition and employer wellbeing practice and provision.

As with many studies that explore workplace wellbeing support, we did not utilize an a priori theoretical framework in our coding and analysis of data. However, when considering our findings theoretically, various frameworks are of relevance, for example, institutional theory for understanding the influences on SMEs’ decisions around wellbeing provision [82] and COM-B for understanding employees’ engagement with employer-provided support [83]. Importantly, in terms of effective implementation of workplace wellbeing initiatives, viewing our findings through the lens of the Consolidated Framework for Implementation Research (CFIR) may be particularly useful to local authorities, SMEs, and others wishing to understand how best to encourage uptake and engagement. CFIR is an overarching framework useful for guiding the implementation of health initiatives and is helpful for framing our findings—particularly around barriers and facilitators—in terms of their application to implementation of workplace wellbeing agendas and initiatives. Studies into the implementation of wellbeing initiatives have utilized CFIR at both organizational (e.g., exploring SME implementation of workplace wellbeing activities [42,84]), and individual (e.g., exploring engagement of employees with wellbeing initiatives [85]) levels. The framework may be usefully applied to our findings, as our study spans the engagement with available workplace health and wellbeing provision of both SME organizations and employees, and the relevant barriers and facilitators.

CFIR consists of five domains: intervention characteristics, outer setting, inner setting, individuals involved, and implementation process [86]. Many of the themes identified in our qualitative analysis map onto the CFIR framework, as do our integrated mixed-methods findings and several of the barriers and facilitators identified in Figure 3. For instance, at the level of the intervention (for example, the support available to SMEs via the local authority), CFIR emphasizes the importance of factors such as financial cost and the importance of intervention adaptation to ensure appropriate fit with the organization. These are reflected in our findings, for example, around the need to tailor support to the diverse requirements of SMEs and SME concerns about organizational burden. ‘Outer setting’ relates to the political, economic, and social context, such as the economic environment, and our findings suggest that the economic environment, including policies and legal requirements, were among the drivers and barriers to SME provision.

Inner setting factors, which related to organizational characteristics, were particularly apparent in our analysis, with factors such as size of organization, resources to support workplace wellbeing, and degree of prioritization of workplace wellbeing support, identified as barriers or facilitators to SME provision. The CFIR domain of ‘individuals involved’ was also prominent in our analysis, as reflected in our findings about the role of beliefs and values in influencing SMEs and employees’ uptake of support, and the importance of workplace ‘champions’ in driving SME engagement and provision. Overall, our findings also lend support to the importance of the planning and implementation process domain, which emphasizes the importance of successful implementation of factors such as needs assessment, strategic planning, and engagement processes. CFIR may be a useful framework for those wishing to effectively implement wellbeing provision at the local authority or organizational levels, and when viewed in tandem with our findings, provides a useful guide for action to address workplace wellbeing in SMEs.

### Strengths and Limitations

A strength of the study is the focus on barriers and facilitators to uptake and provision of workplace health and wellbeing support at both organizational and employee levels, enabling us to explore the commonalities, differences and interplay between these. In addition, the integration of mixed methods provided both breadth and depth of analysis and generated some important additions to knowledge.

A limitation of the study is its focus on a single urban geographical area, meaning that some findings may be particular to that specific local context and its industries. However, the rich qualitative data, descriptions and analyses do allow for ‘naturalistic generalization’ and ‘transferability’, where stakeholders such as SMEs, local authorities, or employees, are invited to apply findings to their own contexts and situations [87]. The study also purposely focused on a subset of SMEs—those with between 10 and 249 employees. The vast majority of SMEs in the United Kingdom are smaller, ‘micro’ organizations with fewer than 10 employees, and their experiences of workplace wellbeing provision and uptake are likely to differ from those of the organizations represented in this study. Further research is needed to understand the experience and support needs of micro-SMEs and those that work within them.

## 8. Conclusions

Workplaces offer a useful route through which to impact the health and wellbeing of working age people and SMEs employ a significant proportion of the workforce. However, encouraging SMEs and their employees to engage with wellbeing agendas can be challenging. Our study identified a range of factors that act as barriers to SME engagement with workplace wellbeing initiatives and SME wellbeing provision and impede employee uptake of employer-provided provision. Alongside these factors, we provide valuable insights into how organizational and employee engagement with wellbeing initiatives might be encouraged and facilitated.

Our study found evidence of effective workplace wellbeing practice from the local authority and its partner organizations (including public health) and SMEs, but also scope for development and improvement. As local authorities, public health teams and SMEs attempt to address the enduring issue of effectively supporting employee wellbeing, our findings provide a valuable basis for developing strategies that can improve workforce health and wellbeing and potentially improve productivity. Trust, awareness, knowledge, and communication may be particularly valuable areas of focus.

## Figures and Tables

**Table 1:** SME survey respondents’ characteristics compared with the local authority area.

Sector	SMEs (10–249) in Sample		SMEs (10–249) in Local Authority Area *
	Number	Percentage	Percentage
Human health and social work activities	20	19	12
Accommodation and food service activities	4	4	4
Transportation and storage	2	2	4
Manufacturing	19	18	26
Wholesale and Retail trade	9	9	20
Education	7	7	2
Construction	5	5	7
Professional, scientific, and technical activities	7	7	7
Other	30	29	18
**Size**	**SMEs (10–249) in Sample**		**SMEs (10–249) in Local Authority Area**
	**Number**	**Percentage**	**Percentage (estimated)**
10–49	73	71	82.6
50–99	20	19	11.8
100–249	10	10	5.6

* Based on UK Office for National Statistics SME Profile Estimates for the Local Authority Area (2021).

**Table 2:** Number of participants recruited via each method.

Recruitment Method	Number of Participants
Following survey completion	2
Via stakeholder contact lists	2
Via attendance at business events	1
Snowball sampling	1
Commercially available list	2

**Table 3:** Overview of questionnaire topic areas.

Topic Area	Description
Background information	Included questions about SME size, sector, time in operation, staff composition and working patterns.
SME engagement with available workplace health and wellbeing support	Explored SME engagement and reasons for non-engagement, with four wellbeing support services available: the main WHP offered by the council; a regional accreditation scheme; health assessments/NHS health checks; and wellbeing workshops.
SME workplace health and wellbeing practice and wellbeing provision for employees	Questions about SME workplace health and wellbeing practices, including the existence of a workplace health and wellbeing strategy/plan, types of provision available to employees via their employers and resources in place within SMEs to support employee wellbeing (e.g., strategy documentation or dedicated staffing).
The SMEs’ workplace health and wellbeing support needs *	Questions regarding SMEs’ need for different types of health and wellbeing support.
SME’s views and attitudes about workplace health and wellbeing	A 10-item scale exploring SMEs’ attitudes towards workplace health and wellbeing, developed following a literature review of organizational attitudes towards workplace health and wellbeing. Although not exhaustive, the scale aimed to provide coverage of key aspects of employers’ attitudes highlighted in the literature (e.g., Pescud et al., 2015 [10]).
Other comments	Opportunity to provide additional comments.

* Detailed analysis of survey findings on SMEs’ workplace health and wellbeing support needs is not included in this paper. This information was primarily used to inform recommendations for the local authority about future workplace health and wellbeing support for SMEs.

**Table 4:** Role of SME representatives who completed the survey.

Position in SME	Number
Business owner/proprietor	14
HR director/manager/lead	14
Senior manager (non-HR)	42
Other	33

**Table 5:** Sector and size of SMEs that participated in interviews.

Sector	Number of SMEs
Human health and social work activities	2
Accommodation and food service activities	1
Transportation and storage	1
Manufacturing	2
Wholesale and Retail trade	1
Other	1
**Size**	**Number of SMEs**
10–49	4
50–99	2
100–149	1
150–199	0
200–249	1

**Table 6:** Demographic information on SME employee participants.

Demographic Category	Frequency
**Gender**	
Male	4
Female	4
**Age**	
18–24	1
25–34	3
35–44	2
45–54	1
55–64	0
65+	0
Not provided	1
**Ethnicity**	
Asian or Asian British	2
Black, African, Caribbean or Black British	1
White British	4
Not provided	1
**Size of employing organization**	
10–49	4
50–99	1
100–149	1
150–199	0
200–249	1
Don’t know/unsure	1
**Industry sector of employing organization**	
Construction	1
Transport and storage	1
Education	1
Human health and social work activities	3
Manufacturing	1
Administrative and support service activities	1

**Table 7:** Explanatory variables in the quantitative analyses.

Variable	Categories
Number of employees	Less than 50/50 or more
More than 50% of employees on casual contracts	Yes/No
More than 50% of employees on part-time contracts	Yes/No
Time trading (years)	More than 10/less than 10
Category of enterprise	Manufacturing or construction/Other
Person or department with specific responsibility for promoting staff health	Yes/No
Recognized trade union in place	Yes/No
More than half of employees men	Yes/No
More than half of employees undertaking manual/routine work	Yes/No
More than half of employees from a minority ethnic background	Yes/No

**Table 8:** Outline of framework analytic process.

Framework Stage	Brief Description
1. Familiarisation with data	N.L. and I.F. read through a selection of interview and focus group transcripts.
2. Coding	Deductive coding based on evaluation research questions, aims, focus group and interview schedules to develop an initial list of codes. Meetings to discuss and agree codes. Ongoing, inductive coding of additional transcripts alongside deductive coding.
3. Development of analytical framework	N.L. and I.F. developed an initial codebook. Codebook agreed by other team members. N.L. and I.F. further refined the codebook at meetings. Two members of the PHIRST Connect Public Involvement in Research Group (PIRg) contributed to analysis (A.D.-P. and J.J. (John Jackson)). A.D.-P. supported the development of the codebook used for SME employees. N.L. and I.F. continued to review transcripts using the most recent version/s of the codebook and reached consensus on a final codebook incorporating considerations made from the PIRg members.
4. Applying analytical framework	N.L. and I.F. coded all transcripts using the final codebook, ensuring it had been used on all data. J.J. (John Jackson) contributed to analysis by coding a section of an SME employer interview transcript. To ensure consistency N.L. and I.F. held regular meetings and daily communication to discuss codes and reach consensus.
5. Charting	N.L. and I.F. conducted a charting process, led by N.L. Microsoft Excel spreadsheets were used to create a framework matrix for interviews and focus groups. Qualitative responses to final ‘other comments’ section of the questionnaire (16 responses) were incorporated into the framework matrix by I.F., N.L. and I.F. met to confirm agreement.
6. Mapping & interpretation	N.L. led on mapping and interpretation process which commenced with examination of the framework matrix for potential themes. N.L. and I.F. systematically explored the matrix, identifying themes within and across framework categories. N.L. and I.F. worked together through regular meetings to refine and reach consensus on themes. Refined themes were reviewed by all members of the team including PIRg members, A.D.-P. and J.J. (John Jackson), prior to additional consolidation of refined themes conducted by N.L. and I.F.

**Table 9:** Sources of support used in the previous 12 months, n (%).

	WHP	Regional Accreditation Scheme	Health Assessments/NHS Checks	Wellbeing Workshops
Yes	5 (4.9)	2 (1.9)	16 (15.5)	9 (8.7)
No	91 (88.3)	90 (87.4)	75 (72.8)	86 (83.5)
Don’t know	7 (6.8)	11 (10.7)	12 (11.2)	8 (7.8)
Total	103	103	103	103

**Table 10:** Reasons for not accessing support, n (%) *.

	WHP	Regional Accreditation Scheme	Health Assessments/NHS Checks	Wellbeing Workshops
Did access support	5 (4.9)	2 (1.9)	16 (15.5)	9 (8.7)
Do not need	31 (30.10)	19 (18.4)	32 (31.1)	36 (35.0)
Not aware	61 (59.2)	66 (64.1)	40 (38.8)	40 (38.8)
Not sure what support would involve	2 (1.9)	2 (1.9)	2 (1.9)	2 (1.9)
Not enough time	1 (1.0)	1 (1.0)	1 (1.0)	5 (4.9)
Other reason	8 (7.8)	7 (6.8)	8 (7.8)	11 (10.7)
Not sure/don’t know	7 (6.8)	11 (10.7)	12 (11.7)	8 (7.8)
Total	103	103	103	103

* Percentages sum to greater than 100 as more than one reason could be chosen.

**Table 11:** Existence of a workplace health and wellbeing strategy or plan, adjusted for part-time working, time trading, person responsible for staff health/wellbeing, trade union, proportion of employees that were male, and trading sector. An odds ratio of more than 1.0 indicates that a plan is more likely to be in place.

Explanatory Variable	Odds Ratio	95% Confidence Interval	*p*-Value
More than 50% of employees on part-time contracts	1.6	0.33–7.83	0.559
Time trading more than 10 years	5.44	0.95–31.28	0.058
Person with specific responsibility for promoting staff health and wellbeing	2.76	0.87–8.74	0.085
Recognized trade union	2.14	0.32–14.52	0.436
Employees more than 50% male	0.32	0.08–1.21	0.093
SME in manufacturing or construction sectors	0.42	0.10–1.81	0.244

**Table 12:** Consultation of employees about their health and wellbeing needs, adjusted for having a person or department responsible for promoting staff health and wellbeing, employees more than 50% male, and trading sector. An odds ratio of more than 1.0 indicates that consultation is more likely to be undertaken.

Explanatory Variable	Odds Ratio	95% Confidence Interval	*p*-Value
Person with specific responsibility for promoting staff health and wellbeing	5.77	1.50–22.12	0.011
Employees more than 50% male	0.32	0.08–1.32	0.115
SME in manufacturing or construction sectors	0.42	0.11–1.66	0.216

**Table 13:** Reported workplace wellbeing provision available to employees via their SME, n (%).

	Health and Safety	Stress Management/Reduction	Mental Health	Injury Prevention
Yes	97 (94.2)	47 (45.6)	66 (64.1)	71 (68.9)
No	5 (4.9)	50 (48.5)	33 (32.0)	29 (28.2)
Don’t know/not sure	1 (1.0)	6 (5.8)	4 (3.9)	3 (2.9)
Total	103	103	103	103
	**Stopping smoking**	**Drugs or alcohol awareness**	**Healthy lifestyles**	**Ageing well**
Yes	14 (13.6)	30 (29.1)	43 (41.7)	17 (16.5)
No	86 (83.5)	69 (67.0)	56 (54.4)	80 (77.7)
Don’t know/not sure	3 (2.9)	4 (3.9)	4 (3.9)	6 (5.8)
Total	103	103	103	103
	**Employee Assistance Programme**	**Financial management/health**	**Caring responsibilities**	**Line management**
Yes	38 (36.9)	30 (29.1)	30 (29.1)	57 (55.3)
No	56 (54.4)	67 (65.0)	69 (67.0)	40 (38.8)
Don’t know/not sure	9 (8.7)	6 (5.8)	4 (3.9)	6 (5.8)
Total	103	103	103	103

**Table 14:** Significant logistic regression associations between SME characteristics and types of wellbeing provision available to employees. An odds ratio of more than 1.0 indicates the degree to which that support is more likely to be offered.

SME Characteristic and Wellbeing Provision	Odds Ratio	95% Confidence Interval	*p*-Value
**Person with specific responsibility for promoting staff health and wellbeing**			
Mental health & wellbeing:			
Stress management	2.77	1.08–7.10	0.034
Mental health	9.75	2.68–35.48	0.001
Broader personal wellbeing:			
Financial management/health	3.18	1.06–9.52	0.015
Caring responsibilities	3.68	1.20–11.31	0.023
Employee Assistance:	4.62	1.44–14.81	0.010
**Recognized trade union**			
Physical health and wellbeing:			
Healthy lifestyles	5.88	1.63–21.26	0.007
Broader personal wellbeing:			
Caring responsibilities	4.04	1.28–12.77	0.017
Employee Assistance:	10.54	1.84–60.27	0.008
**More than 50% of employees on casual contracts**			
Physical health and safety:			
Health and safety	0.10	0.01–0.85	0.034
Injury prevention	0.14	0.03–0.83	0.030

**Table 15:** SMEs’ stated workplace health and wellbeing support needs, n (%).

	Identifying the Health/Wellbeing Needs of Staff	NHS Health Checks/Assessments	Wellbeing Workshops	Conducting a Staff Wellbeing Survey
Yes	50 (48.5)	57 (55.3)	50 (48.5)	41 (39.8)
No	39 (37.9)	34 (33.0)	42 (40.8)	51 (49.5)
Don’t know/not sure	14 (13.6)	12 (11.7)	11 (10.7)	11 (10.7)
Total	103	103	103	103
	**Encourage staff to stop smoking**	**Support staff around drink or drugs**	**Mental health awareness support**	**Healthy ‘behaviour change’ support**
Yes	42 (40.8)	42 (40.8)	58 (56.3)	47 (45.6)
No	52 (50.5)	50 (48.5)	35 (34.0)	46 (44.7)
Don’t know/not sure	9 (8.7)	11 (10.7)	10 (9.7)	10 (9.7)
Total	103	103	103	103
	**Improving workplace health and safety**	**Developing workplace wellbeing policies**		
Yes	37 (35.9)	45 (43.7)		
No	57 (55.3)	48 (46.6)		
Don’t know/not sure	9 (8.7)	10 (9.7)		
Total	103	103		

**Table 16:** Significant multiple logistic regression associations between an SME having fewer than 50 employees and stated workplace health and wellbeing support needs (odds ratio, 95% confidence interval, *p*-value). An odds ratios of less than 1.0 indicates that expressed need of support in that area is less likely.

Support Need	Odds Ratio	95% Confidence Interval	*p*-Value
Identifying the health/wellbeing needs of staff	0.19	0.05–0.72	0.014
Conducting a staff wellbeing survey	0.26	0.08–0.77	0.016
Healthy ‘behaviour change’ support	0.35	0.13–0.98	0.045

**Table 17:** Themes and sub-themes developed through the framework analysis process.

SME Engagement with Workplace Health and Wellbeing Support (four themes)	
Theme	Sub-theme
1. Knowledge and awareness of available support	(a) Overall level of awareness of available support
	(b) Clarity and accessibility of messaging and communications
2. Organizational perceptions and conceptualizations	(a) Conceptualization of workplace health and wellbeing
	(b) Perception of organizational burden
	(c) Perception of organizational need
	(d) Fear of consequences
3. The importance of flexibility	(a) The need for a varied and multi-pronged approach
	(b) Tailoring support to SME needs
4. The importance of partnership and relationships	(a) Joint and collaborative working
	(b) Building trusted relationships between SMEs and those providing services
**SME provision for employees (four themes)**	
**Theme**	**Sub-theme**
1. Sufficiency of available support	(a) Varying levels of provision
	(b) Providing a supportive environment
2. Matching provision to employee need	(a) Informality of staff health and wellbeing needs assessments
	(b) Variation in awareness of staff wellbeing needs
	(c) Reactive rather than proactive approach
3. Drivers of SME provision	(a) Business case and the importance of staff to effective business
	(b) Organizational ethos and culture
	(c) Compliance
	(d) Importance of organizational workplace wellbeing ‘champions’
4. Practical issues	(a) Prioritization—balancing wellbeing provision and business sustainability
	(b) Lack of organizational resources
**Employee uptake of employer-provided support (five themes)**	
**Theme**	**Sub-theme**
1. Lack of awareness of the range of employer-provided support	N/A
2. Practical issues	(a) Logistical difficulties—employee working location or role
	(b) The need to timetable workplace wellbeing support within working hours
3. Employees’ reluctance to disclose issues	(a) General reluctance to disclose
	(b) Concerns about confidentiality
	(c) Suspicions regarding employer motivations and fear of negative consequences
4. Organizational culture and approach/environment	(a) Employer focus and prioritization
	(b) The importance of an ‘open’ health and wellbeing culture
5. Staff workplace health and wellbeing beliefs and attitudes	(a) Employees’ reactive/remedial approach to workplace health and wellbeing
	(b) Beliefs about the appropriateness of discussing wellbeing issues

## Data Availability

The participants of this study did not provide written consent for their data to be shared publicly, and the data contain some information that could compromise the privacy of research participants; therefore, supporting data are not publicly available. However, data supporting the findings of this study are available from the corresponding author, NL, upon reasonable request.

## Appendix A. Survey Questionnaire for SMEs

**Survey questionnaire**

This survey is about your SME’s engagement with health and wellbeing support for its staff and how it promotes staff health and wellbeing.

Please only complete the survey if your SME has a base in the [locality removed] and employs between 10 and 250 employees.

Thank you for your participation.

**A. Background information**

**We’d like to start with a few background details so that we can get an idea of the different types of SMEs completing the survey.**

Your business/organisation

**1. Approximately how many people does this SME employ? Please include both full-time and part-time, permanent, temporary, and casual staff. Please do not include contractors or agency staff.**

0–9	
10–49	
50–99	
100–149	
150–199	
200–249	
250 or more	

Responses for ‘0–9’ and ‘250 or more’ automatically directed to the end of the questionnaire

**2. In which area/s in [locality removed] (also known as ward/s) is this SME located (please choose as many as apply)?**

Option 1	
Option 2	
Option 3 etc.	

**3. Are more than 50% of your employees:**

	Yes	No	Not sure
On casual contracts			
On part-time contracts			

**4. What is the approximate annual turnover of this SME?**

Less than £1000	
£1000–£9999	
£10,000–£99,999	
£100,000–£1 million	
£1 million–£10 million	
£10 million–£50 million	
More than £50 million	
Don’t know/not sure	
Rather not say	

**5. How long has this SME been trading/operating?**

Less than one year	
Between one and 5 years	
More than 5 years but less than 10	
More than 10 years	
Don’t know/not sure	

**6. Which of the following best describes this SME? Please choose as many as apply.**

A private limited company (Ltd.)	
A partnership	
A community or voluntary organization	
A registered charity	
Other (please specify)	

**7. What category of industry best describes this SME? Please choose only one option.**

Manufacturing	
Transportation and storage	
Wholesale and retail trade (including repair of motor vehicles and motorcycles)	
Construction	
Accommodation and food service activities	
Administrative and support service activities	
Real estate activities	
Information and communication	
Professional, scientific and technical activities	
Education	
Human health and social work activities	
Arts, entertainment and recreation	
Water supply, sewerage, waste management and remediation activities	
Agriculture, forestry and fishing	
Financial and insurance activities	
Other	

**8. Does this SME have a person or department with specific responsibility for promoting staff health and wellbeing?**

**Yes**	
No	
Don’t know/not sure	

**9. Is there a recognised trade union within this SME?**

Yes	
No	
Don’t know/not sure	
Rather not say	

**Evidence suggests that male employees or staff who are mainly undertaking manual or routine work are less likely to engage in health and wellbeing support. Therefore, some of {locality removed] health and wellbeing support is aimed at SMEs with mainly male employees or where staff are mainly undertaking manual or routine work.**

**10. In your SME:**

	Yes	No	Not sure
Do more than half of employees identify as men			
Do more than half of employees undertake manual or routine work			
Are more than half of employees from a Black, Asian, or Minority Ethnic background			

**We’d like to know a little about your SME’s management team. This is so we can describe the different types of SMEs who have taken part in the survey.**

**11. Would 50% or more of your management team (for example, owner/s and senior management) describe themselves as:**

	Yes	No	Not sure
From a Black, Asian or Minority Ethnic background			
Female			
Living with a disability			
LGBTQ+ (lesbian, gay, bisexual, transgender, queer/questioning, and others)			

**Your details**

**We’d like to get an idea of who within each SME is completing the survey.**

**1. Which of the following best describes your role within this SME? Please choose only one option.**

Business owner/proprietor	
HR director/manager/lead	
Senior manager (non-HR)	
Other	

**B. About this SME’s use of health and wellbeing support**

**1. Different types of free support are available to employers in [locality removed] to help them support the health and wellbeing of their workforce. Has this SME made use of any of the following within the last 12 months? If ‘no’, please indicate why not. Please choose as many as apply.**

	Yes	No, because we do not need this type of support	No, because we were not aware that this support existed	No, because we were not sure what this support would involve	No, because we have not had the time	No, for another reason—please state	Do not know/not sure
WHP							
Regional accreditation scheme							
Health Assessments/NHS Health Checks for staff							
Wellbeing workshops (e.g., stopping smoking, mental wellbeing)							

**2. Did this SME make use of any of the above types of support before the COVID-19 pandemic (i.e., before March 2020).**

Yes	
No	
Don’t know/not sure	
If yes, please say which one/s	

**C. About health and wellbeing support within this SME**

**1. Does this SME:**

	Yes	No	Don’t know/not sure/
Have an agreed workplace health and wellbeing strategy or plan?			
Consult employees about their health and wellbeing needs (e.g., through a survey, needs assessment or at an appraisal)?			

**2. In the last 12 months, has this SME offered support or training for its employees in any of the following areas?**

	Yes	No	Don’t know/not sure/
Health and safety			
Stress management/reduction			
Mental health			
Injury prevention			
Stopping smoking			
Drugs or alcohol awareness			
Healthy lifestyles (e.g., physical activity or healthy eating)			
Ageing well			
An employee assistance programme (EAP)			
Financial management/health			
Managing caring responsibilities			
Line management			

**3. In the past 12 months, has this SME made use of any free or paid for health and wellbeing support/services from any external organisation/provider?**

Yes	
No	
Don’t know/not sure	

** **

**If yes, was this via (tick all that apply):**	
Local authority	
A private organisation	
A charitable or voluntary organisation	
NHS	
Other (please state)	

**D. Your SME’s health and wellbeing support needs**

**1. Do you think your SME would be interested in any of the following types of health and wellbeing support for its workforce?**

	Yes	No
Support with identifying health/wellbeing needs of staff		
NHS Health Checks/Assessments for staff		
Wellbeing workshops for staff (e.g., stopping smoking, mental wellbeing)		
Help with conducting a staff wellbeing survey		
Support/advice to encourage staff to stop smoking		
Support/advice to support staff around drink or drugs		
Mental health awareness support for staff		
Healthy ‘behaviour change’ support for staff		
Support with improving workplace health and safety		
Support to develop workplace wellbeing policies		

** **

If there are any other types of health and wellbeing support this SME would be interested in, please give details here.

**E. Your SME’s views about workplace health and wellbeing**

**1. We’d like to find out a little about this SME’s views of workplace health and wellbeing. Please read the following statements and indicate how much you agree or disagree with each. As far as possible, please answer in terms of the views held by your business/organisation, rather than giving your personal views.**

**There are no right or wrong answers and your answers will remain anonymous. (Please choose only one option per row)**

	Strongly agree	Agree	Neither agree nor disagree	Disagree	Strongly disagree
Employers have a responsibility to support the health and wellbeing of employees.					
The financial costs of supporting employee health and wellbeing outweigh the benefits.					
The time and effort involved in supporting staff health and wellbeing are well worth it.					
There is a direct link between work and employees’ health and wellbeing.					
Businesses of all sizes should allocate resources towards supporting employees’ health and wellbeing.					
The health and wellbeing of employees should be a top priority for businesses.					
Better workforce health and wellbeing can improve productivity.					
Most staff have health and wellbeing needs that their employers can help with.					
All employers should have a clear plan for supporting staff health and wellbeing.					
Employers should have regular discussions with their employees about their health and wellbeing needs.					

**F. Other comments about workplace health and wellbeing**

**1. If there is anything else you would like to say about workplace health and wellbeing in this SME, please give your comments in the box below.**

## References

[1] Office for National Statistics (2023). Time Use in the UK: March 2023. https://www.ons.gov.uk/peoplepopulationandcommunity/personalandhouseholdfinances/incomeandwealth/bulletins/timeuseintheuk/march2023.

[2] U.S. Bureau of Labor Statistics Average Hours Per Day Spent in Selected Activities on Days Worked by Employment Status and Sex. https://www.bls.gov/charts/american-time-use/activity-by-work.htm.

[3] Danna K., Griffin R.W. (1999). Health and Well-Being in the Workplace: A Review and Synthesis of the Literature. J. Manag..

[4] Guest D.E. (2017). Human Resource Management and Employee Well-Being: Towards A New Analytic Framework.

[5] Sorensen G., Dennerlein J.T., Peters S.E., Sabbath E.L., Kelly E.L., Wagner G.R. (2021). The future of research on work, safety, health and wellbeing: A guiding conceptual framework. Soc. Sci. Med..

[6] Peters S.E., Dennerlein J.T., Wagner G.R., Sorensen G. (2022). Work and Worker Health in the Post-Pandemic World: A Public Health Perspective. Lancet Public Health.

[7] World Health Organisation Cardiovascular Diseases (CVDs). https://www.who.int/news-room/fact-sheets/detail/cardiovascular-diseases-(cvds).

[8] Litchfield P., Cooper C., Hancock C., Watt P. (2016). Work and wellbeing in the 21st century. Int. J. Environ. Res. Public Health.

[9] Lee N.K., Roche A., Duraisingam V., Fischer J.A., Cameron J. (2014). Effective interventions for mental health in male-dominated workplaces. Ment. Health Rev. J..

[10] Pescud M., Teal R., Shilton T., Slevin T., Ledger M., Waterworth P., Rosenberg M. (2015). Employers’ views on the promotion of workplace health and wellbeing: A qualitative study. BMC Public Health.

[11] Office for Health Improvement & Disparities Health Disparities and Health Inequalities: Applying All Our Health. https://www.gov.uk/government/publications/health-disparities-and-health-inequalities-applying-all-our-health/health-disparities-and-health-inequalities-applying-all-our-health.

[12] World Health Organization Breaking Barriers Towards More Equitable Health Systems for Everyone. https://www.who.int/activities/breaking-barriers-towards-more-equitable-health-systems-for-everyone.

[13] World Health Organisation (2022). Global Spending on Health Rising to the Pandemic’s Challenges.

[14] Stoewen D.L. (2017). Dimensions of wellness: Change your habits, change your life. Can. Vet. J..

[15] Pronk N.P., Kleinman D.V., Richmond T.S. (2021). Healthy People 2030: Moving toward equitable health and well-being in the United States. EClinicalMedicine.

[16] Eurostat How Many Hours Do EUROPEANS Work Per Week?—Products Eurostat News—Eurostat. https://ec.europa.eu/eurostat/web/products-eurostat-news/-/ddn-20180125-1.

[17] Cooper C., Cartwright S. (2024). Healthy Mind; Healthy Organization—A Proactive Approach to Occupational Stress. Hum. Relat..

[18] Lawson G., Haggar T., Hewlett K., Hall S., Piggott H., Hesketh R., Regan Z., Wojciechowska M., Dacombe R., Morgan C. (2023). Experiencing the Cost-of-Living Crisis: The Impact on Mental Health.

[19] Cribb J., Waters T. (2024). Institute for Fiscal Studies Seven Key Facts about UK Living Standards IFS Report R315. https://ifs.org.uk/publications/seven-key-facts-about-uk-living-standards.

[20] Florisson R. (2024). The UK Insecure Work Index 2024.

[21] Office for National Statistics (2023). Sickness Absence in the UK Labour Market: 2022 Sickness Absence Rates of Workers in the UK Labour market, Including Number of Days Lost and Reasons for Absence.

[22] Health and Safety Executive (2024). Costs to Britain of Workplace Fatalities and Self-Reported Injuries and Ill Health, 2022/23. https://www.hse.gov.uk/statistics/assets/docs/cost-to-britain.pdf.

[23] Health and Safety Executive Working Days Lost in Great Britain. https://www.hse.gov.uk/statistics/dayslost.htm.

[24] UK Parliament Economic Update: Inactivity due to Illness Reaches Record. https://commonslibrary.parliament.uk/economic-update-inactivity-due-to-illness-reaches-record/.

[25] Atwell S., Vriend M., Finch D., Mooney A., Rocks C., Bibby J. (2024). How Can the Next Government Improve the Health of the Workforce and Boost Growth.

[26] Hassan E., Austin C., Celia C., Disley E., Hunt P., Marjanovic S., Ala’a Shehabi van Dijk L.V., Van Stolk C. (2009). Health and wellbeing at work in the United Kingdom.

[27] Chartered Institute of Personnel and Development (CIPD) (2018). Health and Well-Being at Work. https://healthwellbeingwork.co.uk/.

[28] Camisa V., Gilardi F., Di Brino E., Santoro A., Vinci M.R., Sannino S., Bianchi N., Mesolella V., Macina N., Focarelli M. (2020). Return on investment (ROI) and development of a workplace disability management program in a hospital—A pilot evaluation study. Int. J. Environ. Res. Public Health.

[29] Deloitte (2022). Mental Health and Employers The Case for Investment-Pandemic and Beyond. https://www.deloitte.com/uk/en/services/consulting/analysis/mental-health-and-employers-the-case-for-investment.html.

[30] Pieper C., Schröer S., Eilerts A.L. (2019). Evidence of workplace interventions-A systematic review of systematic reviews. Int. J. Environ. Res. Public Health.

[31] Shiri R., Nikunlaakso R., Laitinen J. (2023). Effectiveness of Workplace Interventions to Improve Health and Well-Being of Health and Social Service Workers: A Narrative Review of Randomised Controlled Trials. Healthcare.

[32] Suter J., Kowalski T., Townley B. (2021). Management Perspectives Workforce Wellbeing: Supporting Small Businesses. https://www.york.ac.uk/business-society/research/management/policy/archive/workforce_wellbeing_north_yorkshire/.

[33] Department for International Trade (2020). Small and Medium-Sized Enterprises Action Plan 2020 to 2022. https://assets.publishing.service.gov.uk/government/uploads/system/uploads/attachment_data/file/961722/SME-Action-Plan.pdf.

[34] Department for Business and Trade Small Business Survey 2022: Businesses with Employees. https://www.gov.uk/government/statistics/small-business-survey-2022-businesses-with-employees.

[35] World Bank SME Finance. https://www.worldbank.org/en/topic/smefinance.

[36] Department for Business & Trade Business Population Estimates for the UK and Regions 2023: Statistical Release—GOV.UK. https://www.gov.uk/government/statistics/business-population-estimates-2023/business-population-estimates-for-the-uk-and-regions-2023-statistical-release.

[37] Martin A., Kilpatrick M., Scott J., Cocker F., Dawkins S., Brough P., Sanderson K. (2020). Protecting the Mental Health of Small-to-Medium Enterprise Owners: A Randomized Control Trial Evaluating a Self-Administered Versus Telephone Supported Intervention. J. Occup. Environ. Med..

[38] Lai Y., Saridakis G., Blackburn R. (2015). Job stress in the United Kingdom: Are small and medium-sized enterprises and large enterprises different?. Stress Health.

[39] Micheli G.J.L., Cagno E. (2010). Dealing with SMEs as a whole in OHS issues: Warnings from empirical evidence. Saf. Sci..

[40] Cocker F., Martin A., Scott J., Venn A., Sanderson K. (2013). Psychological distress, related work attendance, and productivity loss in small-to-medium enterprise owner/managers. Int. J. Environ. Res. Public Health.

[41] Blake H., Bullock H., Chouliara N. (2023). Enablers and barriers to mental health initiatives in construction SMEs. Occup. Med..

[42] Saito J., Odawara M., Takahashi H., Fujimori M., Yaguchi-Saito A., Inoue M., Uchitomi Y., Shimazu T. (2022). Barriers and facilitative factors in the implementation of workplace health promotion activities in small and medium-sized enterprises: A qualitative study. Implement. Sci. Commun..

[43] Hannon P.A., Garson G., Harris J.R., Hammerback K., Sopher C.J., Clegg-Thorp C. (2012). Workplace health promotion implementation, readiness, and capacity among midsize employers in low-wage industries: A national survey. J. Occup. Environ. Med..

[44] Lindström K.J. (2004). Work organization interventions in small and medium-sized enterprises in Scandinavia; Commentary IV. Soz. Praventivmed..

[45] Office for National Statistics 2021 Census Profile for Areas in England and Wales. https://www.nomisweb.co.uk/sources/census_2021/report.

[46] El-Osta A., Altalib S., Alaa A., Al-Ammouri M., Riboli-Sasco E., Majeed A., Kudrna K. (2024). A scoping review of UK local government workplace health and wellbeing programmes, 18 June 2024, PREPRINT (Version 1).

[47] NHS NHS Health Check. https://www.nhs.uk/conditions/nhs-health-check/.

[48] Creswell J.W., Plano-Clark V.L. (2017). Choosing a Mixed Methods Design.

[49] Tariq S., Woodman J. (2013). Using mixed methods in health research. JRSM Short Rep..

[50] Denzin N., Lincoln Y. (2018). The Sage Handbook of Qualitative Research.

[51] Fetters M.D., Curry L.A., Creswell J.W. (2013). Achieving integration in mixed methods designs—Principles and practices. Health Serv. Res..

[52] Brannen J. (2005). Mixing methods: The entry of qualitative and quantitative approaches into the research process. Int. J. Soc. Res. Methodol. Theory Pract..

[53] Tong A., Sainsbury P., Craig J. (2007). Consolidated criteria for reporting qualitative research (COREQ): A 32-item checklist for interviews and focus groups. Int. J. Qual. Health Care.

[54] Harris P.A., Taylor R., Thielke R., Payne J., Gonzalez N., Conde J.G. (2009). Research electronic data capture (REDCap)-A metadata-driven methodology and workflow process for providing translational research informatics support. J. Biomed. Inform..

[55] Sahlqvist S., Song Y., Bull F., Adams E., Preston J., Ogilvie D. (2011). Effect of questionnaire length, personalisation and reminder type on response rate to a complex postal survey: Randomised controlled trial. BMC Med. Res. Methodol..

[56] Devers K.J., Frankel R.M. (2000). Study Design in Qualitative Research-2: Sampling and Data Collection Strategies. Educ. Health.

[57] Long J.S. (1997). Regression Models for Categorical and Limited Dependent Variables.

[58] Hosmer D., Lemeshow S., Sturdivant R. (2013). Applied Logistic Regression.

[59] Pregibon D. (1980). Goodness of link tests for generalized linear models. Appl Stat.

[60] Stata Corp (2017). Stata Statistical Software: Release 15.

[61] Gale N.K., Heath G., Cameron E., Rashid S., Redwood S. (2013). Using the framework method for the analysis of qualitative data in multi-disciplinary health research. BMC Med. Res. Methodol..

[62] Midgley N., Parkinson S., Holmes J., Stapley E., Eatough V., Target M. (2015). Beyond a diagnosis: The experience of depression among clinically-referred adolescents. J. Adolesc..

[63] Target M., Midgley N. (2016). Framework analysis: A worked example of a study exploring young people’s experiences of depression. Qual. Res. Psychol..

[64] Chartered Institute of Personnel and Development (CIPD) Wellbeing at Work Factsheet. https://www.cipd.org/uk/knowledge/factsheets/well-being-factsheet/.

[65] Department of Work and Pensions (DWP) Sickness Absence and Health in the Workplace: Understanding Employer Behaviour and Practice—An Interim Summary—GOV.UK. https://www.gov.uk/government/publications/sickness-absence-and-health-employer-behaviour-and-practice/sickness-absence-and-health-in-the-workplace-understanding-employer-behaviour-and-practice-an-interim-summary#introduction.

[66] Taylor A.W., Pilkington R., Montgomerie A., Feist H. (2016). The role of business size in assessing the uptake of health promoting workplace initiatives in Australia. BMC Public Health.

[67] Department of Work and Pensions (DWP) Incentivising SME Uptake of Health and Wellbeing Support Schemes—GOV.UK. https://www.gov.uk/government/publications/incentivising-sme-uptake-of-health-and-wellbeing-support-schemes/incentivising-sme-uptake-of-health-and-wellbeing-support-schemes.

[68] Unit O.D., Durban R., Director R.G.C. (2022). National Institute for Health and Care Excellence. Mental Wellbeing at Work. Evidence Review F: Barriers and Facilitators to the Implementation and Delivery of Interventions to Improve and Protect Mental Wellbeing at Work.

[69] Phillips K., Stokols D., McMahan S., Grzywacz J.G. (2004). Strategies for Health Promotion in Small Businesses by Strategies for Health Promotion in Small Businesses. Am. J. Health Promot..

[70] McCoy K., Stinson K., Scott K., Tenney L., Newman L.S. (2014). Health promotion in small business: A systematic review of factors influencing adoption and effectiveness of worksite wellness programs. J. Occup. Environ. Med..

[71] Benning F.E., van Oostrom S.H., van Nassau F., Schaap R., Anema J.R., Proper K.I. (2022). The Implementation of Preventive Health Measures in Small and Medium-Sized Enterprises—A Combined Quantitative/Qualitative Study of Its Determinants from the Perspective of Enterprise Representatives. Int. J. Environ. Res. Public Health.

[72] Coppens E., Hogg B., Greiner B.A., Paterson C., de Winter L., Mathieu S., Cresswell-Smith J., Aust B., Leduc C., Van Audenhove C. (2023). Promoting employee wellbeing and preventing non-clinical mental health problems in the workplace: A preparatory consultation survey. J. Occup. Med. Toxicol..

[73] Nowrouzi B., Gohar B., Nowrouzi-Kia B., Garbaczewska M., Chapovalov O., Myette-Côté É., Carter L. (2016). Facilitators and barriers to occupational health and safety in small and medium-sized enterprises: A descriptive exploratory study in Ontario, Canada. Int. J. Occup. Saf. Ergon..

[74] Spence G.B. (2015). Workplace wellbeing programs: If you build it they may NOT come…because it’s not what they really need!. Int. J. Wellbeing.

[75] Quirk H., Crank H., Carter A., Leahy H., Copeland R.J. (2018). Barriers and facilitators to implementing workplace health and wellbeing services in the NHS from the perspective of senior leaders and wellbeing practitioners: A qualitative study. BMC Public Health.

[76] Mellor N., Webster J. (2013). Enablers and challenges in implementing a comprehensive workplace health and well-being approach. Int. J. Workplace Health Manag..

[77] Williams S.J., Snow D.M. (2012). Promoting health in small and medium-sized enterprises. J. Small Bus. Enterp. Dev..

[78] Hynes J., Crooke B. (2024). Perceived Drivers of Engagement and Disengagement in Workplace Wellbeing Programmes; Qualitative Evidence from Employees in the Republic of Ireland. Humanist. Manag. J..

[79] Paterson C., Leduc C., Maxwell M., Aust B., Strachan H., O’Connor A., Tsantila F., Cresswell-Smith J., Purebl G., Winter L. (2024). Barriers and facilitators to implementing workplace interventions to promote mental health: Qualitative evidence synthesis. Syst. Rev..

[80] Wels J. (2020). The role of labour unions in explaining workers’ mental and physical health in Great Britain. A longitudinal approach. Soc. Sci. Med..

[81] Kromydas T., Demou E., Leyland A.H., Katikireddi S.V., Wels J. (2023). Effect of trade unions on the mental health of UK workers before and during the COVID-19 pandemic: A longitudinal analysis using Understanding Society data. Lancet.

[82] Scott W.R. (1995). Institutions and Organizations.

[83] Michie S., van Stralen M.M., West R. (2011). The behaviour change wheel: A new method for characterising and designing behaviour change interventions. Implement. Sci..

[84] Campmans J., Smit D., van Oostrom S., Engels J., Proper K. (2023). Barriers and facilitators to the implementation of workplace health promotion programs: Employers’ perceptions. Front. Public Health.

[85] Smit D.J.M., Proper K.I., Engels J.A., Campmans J.M.D., van Oostrom S.H. (2023). Barriers and facilitators for participation in workplace health promotion programs: Results from peer-to-peer interviews among employees. Int. Arch. Occup. Environ. Health.

[86] Damschroder L.J., Aron D.C., Keith R.E., Kirsh S.R., Alexander J.A., Lowery J.C. (2009). Fostering implementation of health services research findings into practice: A consolidated framework for advancing implementation science. Implement. Sci..

[87] Tracy S.J. (2010). Qualitative quality: Eight a ”big-tent” criteria for excellent qualitative research. Qual. Inq..

